# High-throughput proteomics fiber typing (ProFiT) for comprehensive characterization of single skeletal muscle fibers

**DOI:** 10.1186/s13395-020-00226-5

**Published:** 2020-03-23

**Authors:** Sebastian Kallabis, Lena Abraham, Stefan Müller, Verena Dzialas, Clara Türk, Janica Lea Wiederstein, Theresa Bock, Hendrik Nolte, Leonardo Nogara, Bert Blaauw, Thomas Braun, Marcus Krüger

**Affiliations:** 1 CECAD Research Center, Institute for Genetics, University of Cologne, 50931 Cologne, Germany; 2 Max Planck Institute for the Biology of Aging, 50931 Cologne, Germany; 3 Venetian Institute of Molecular Medicine (VIMM), Via Orus 2, 35129 Padova, Italy; 4 Max Planck Institute for Heart and Lung Research, 61231 Bad Nauheim, Germany; 5 Center for Molecular Medicine (CMMC), University of Cologne, 50931 Cologne, Germany

**Keywords:** Muscle fiber proteomics, MyHC profiling, Protein turnover, Phosphoproteomics

## Abstract

**Background:**

Skeletal muscles are composed of a heterogeneous collection of fiber types with different physiological adaption in response to a stimulus and disease-related conditions. Each fiber has a specific molecular expression of myosin heavy chain molecules (MyHC). So far, MyHCs are currently the best marker proteins for characterization of individual fiber types, and several proteome profiling studies have helped to dissect the molecular signature of whole muscles and individual fibers.

**Methods:**

Herein, we describe a mass spectrometric workflow to measure skeletal muscle fiber type-specific proteomes. To bypass the limited quantities of protein in single fibers, we developed a Proteomics high-throughput fiber typing (ProFiT) approach enabling profiling of MyHC in single fibers. Aliquots of protein extracts from separated muscle fibers were subjected to capillary LC-MS gradients to profile MyHC isoforms in a 96-well format. Muscle fibers with the same MyHC protein expression were pooled and subjected to proteomic, pulsed-SILAC, and phosphoproteomic analysis.

**Results:**

Our fiber type-specific quantitative proteome analysis confirmed the distribution of fiber types in the soleus muscle, substantiates metabolic adaptions in oxidative and glycolytic fibers, and highlighted significant differences between the proteomes of type IIb fibers from different muscle groups, including a differential expression of desmin and actinin-3. A detailed map of the Lys-6 incorporation rates in muscle fibers showed an increased turnover of slow fibers compared to fast fibers. In addition, labeling of mitochondrial respiratory chain complexes revealed a broad range of Lys-6 incorporation rates, depending on the localization of the subunits within distinct complexes.

**Conclusion:**

Overall, the ProFiT approach provides a versatile tool to rapidly characterize muscle fibers and obtain fiber-specific proteomes for different muscle groups.

## Background

Skeletal muscles are essential for the maintenance of metabolism throughout the body and act as the force generator for physical movement. Anatomically, skeletal muscles are composed of large numbers of cylindrically shaped muscle fibers with numerous nuclei. Each fiber can be subdivided into myofibrils containing thick and thin myofilaments [1]. The molecular interaction of actin and myosin proteins forms the basis for skeletal muscle contraction and is described as the myosin swinging cross-bridge model [2, 3]. The identification of different myosin heavy chain isoforms (MyHC) represented a breakthrough in the molecular understanding of muscle fibers and has enabled a better understanding of the molecular heterogeneity of skeletal muscles [4, 5]. Several biochemical methods, including protein electrophoresis, ATPase activity assays, and immunostaining, have been used to distinguish and characterize skeletal muscle fibers [6]. For example, slow fibers (type I) are defined by MYH7 expression, whereas the three fast-twitch fibers (IIa, IIb, IIx) express MYH2, MYH4, and MYH1, respectively. In terms of metabolic activity, type I and type IIa fibers are more oxidative, while IIb fibers are described as glycolytic fibers. Type IIx fibers represent intermediate metabolic characteristics and contractile properties, highly dependent on their localization within a muscle [7]. In addition to pure fibers expressing only one MyHC isoform, a number of mixed fibers—including type I/IIa, type IIa/IIx, and type IIb/IIx—contribute to the variability of muscle fibers in skeletal muscle tissues. Fiber type-specific protein expression is also observed for other muscle proteins. Moreover, different fiber types even exhibit distinct numbers and adaptions to whole cellular compartments, such as mitochondria. For example, troponin, tropomyosin, and the calcium pump SERCA exist as slow and fast isoforms, which affect the performance and contractility of fiber types [5]. Due to their varied properties, each muscle fiber type responds differently to external stimuli, including exercise, starvation, or loss of neuronal innervation. The rates of protein synthesis and degradation are also important factors that modulate the physiological activity of muscles. Previous studies based on the uptake of radioactively labeled molecules—such as ^14^C-labeled α-aminoisobutyric acid (AIB) or ^3^H-phenylalanine—revealed elevated protein turnover in muscles containing more oxidative fibers compared to glycolytic muscles [8, 9]. However, the individual proteins that contribute to the uptake of tracers and tissue turnover remained largely unclear. More recently, the analysis of relative incorporation rates of SILAC amino acids such as ^13^C_6_ lysine (Lys-6) has been demonstrated to be a versatile tool for monitoring protein incorporation rates in cell culture [10] and living animals [11, 12]. Hence, a pulsed in vivo SILAC approach may also help to elucidate the turnover of proteins in different skeletal muscle fibers.

Here, we analyzed slow and fast fibers from different skeletal muscle groups in mice and demonstrated that our ProFit approach enables the analysis of fiber type-specific proteomes and phosphoproteomes. A pulsed SILAC approach in living animals revealed different Lys-6 incorporation rates between slow and fast fibers and unraveled protein dynamics in mitochondrial OXPHOS complexes and its assembly factors. In addition, fiber type-specific proteome analysis revealed several differently regulated proteins.

## Methods

### ProFiT workflow

#### Muscle fiber extraction and lysis

Single muscle fibers were isolated using a modified version for single myofiber isolation protocols [13, 14]. In brief, hindlimb skeletal muscles from adult wild-type (C57BL/J6) were dissected and placed in 4 ml DMEM buffer containing 0.2% (w/v) collagenase P and incubated at 37 °C in a thermocycler with gentle agitation. The samples were incubated for ~ 30 min depending on muscle size, age, and condition, and checked regularly until single fibers started to detach from the muscle bundle. Muscles were then transferred into ice-cold DMEM to stop the digestion, and fibers were released by carefully flushing the muscles using a glass Pasteur pipette. Detached fibers were transferred into FACS tubes and then washed three times with ice-cold PBS to remove residual DMEM. Single fibers were collected into 96-well PCR reaction plates (Greiner Bio-One) using a stereomicroscope (Leica), lysed in 4% SDS in PBS, heated for 5 min at 95 °C, and DNA was sheared by sonication for 10 min (Bioruptor) with a 30 s on/off duty cycle. Extracted proteins were reduced and alkylated by adding 10 mM of dithiothreitol (DTT, 30 min at RT) and 40 mM of iodoacetamide (IAA, 20 min at RT in the dark).

#### ProFiT protein digestion

To perform ProFiT, 10% (vol) of the lysed samples were transferred to new 96-well plates. Tryptic digestion was performed using a modified version of the previously described single-pot solid phase-enhanced sample preparation approach [15]. Briefly, 2 μL of prepared Sera-Mag Speed bead stock solution (10 μg/μL, Thermo Scientific) was added to each sample and supplemented with 100% acetonitrile (ACN) in a 1:2 ratio. After thorough mixing, samples were incubated for 8 min and placed on an automated plate washer (BioTek) complemented with a magnetic rack. Magnetic beads were washed twice with 180 μL of 70% ethanol and 180 μL of 100% ACN and then dried by centrifugation for 5 min in a SpeedVac (Eppendorf). Proteins were reconstituted in 5 μL of 50 mM triethylammonium bicarbonate supplemented with 0.1 μg trypsin (Thermo Scientific) and Lys-C (Wako) each. Tryptic digestion was performed overnight at 37 °C. On the following day, peptide-bead mixtures were resuspended, diluted with 100% ACN to a final ACN concentration of > 95%, incubated for 8 min, washed twice in 100% ACN on the automated plate washer, and dried in a SpeedVac. Finally, beads were resuspended in 9 μL of 5% ACN in water, sonicated for 5 min and placed on a magnetic rack. Eluted peptides were recovered into a new 96-well plate and acidified with formic acid before LC-MS/MS.

#### ProFiT LC-MS/MS analysis

Proteomic analysis of ProFiT samples was performed using an Easy nLC II ultra-high performance liquid chromatography (UHPLC) system coupled to an LTQ Orbitrap XL mass spectrometer (both Thermo). Peptides were separated on a 5-cm-long in-house packed column (75 μm ID, 3 μm ReproSil-Pur C18-AQ resins [Dr. Maisch]) using a binary buffer system consisting of buffer A (0.1% FA) and buffer B (0.1% FA in 80% ACN). The separation was achieved by linearly increasing the amount of buffer B from 15 to 28% over 5 min, followed by a linear increase to 45% buffer B over 2 min. Column washing was achieved by increasing the amount of buffer B to 95% for 3 min. The mass spectrometer was set to a resolution of 30,000, an AGC target of 1e6 and a maximum IT of 500 ms during MS acquisition. The top ten most abundant peptide ions were collected with an isolation width of 3 Th and subsequently CID-fragmented and measured in the linear ion trap at a resolution of 7500 with an AGC target of 3e4 and maximum IT of 100 ms.

To further decrease the analysis time per fiber, we developed an alternative method using a Dionex Ultimate 3000 RSLCnano UHPLC system coupled by nanoelectrospray ionization to a Q Exactive HF-X instrument (both Thermo Scientific). Peptides were separated on a 5-cm-long in-house-packed analytical column, filled with 3 μm ReproSil-Pur C18-AQ resins (Dr. Maisch), using a binary buffer system (buffer A = 0.1% FA, buffer B = 80% ACN, 0.1% FA). Samples were loaded onto a trap column at a flow rate of 15 μL/min using the system loading pump. Peptides were transferred onto the analytical column at a flow rate of 1.5 μL/min using a binary high-pressure gradient pump. The gradient was linearly increased from 10 to 50% buffer B over 3 min, then buffer B was increased to 90% within 0.1 min, and the column was washed for 0.5 min. Finally, the column was equilibrated with 10% buffer B within 0.4 min. Full MS spectra were acquired at a resolution of 30,000, an AGC target of 3e6 and maximum IT of 50 ms. The top 20 most abundant peptides were selected for HCD fragmentation (NCE = 30) and measured at a resolution of 15,000, an AGC target of 1e5 and a maximum IT of 22 ms.

#### Data processing, fiber type assignment, and pooling

The RAW data were analyzed using the MaxQuant software (1.5.3.8) [16] and its implemented Andromeda search algorithm [17] using the default settings. The mouse UniProt-FASTA database was used for peptide and protein identification. Peptides for quantification were set to unique to avoid incorrect assignment of non-unique MyHC peptides. As described in [18], skeletal muscle fiber types were assigned by comparing myosin heavy chain (MyHC) isoforms for single fibers. Briefly, the relative amounts of each MyHC isoform were calculated by dividing the intensity of each isoform (MYH1, MYH2, MYH4, MYH7) by the summed intensity of all four isoforms. A fiber was assigned as a type I fiber if the relative quantity of MYH7 was > 80%, as type IIa if MYH2 > 60%, as type IIx if MYH1 > 60%, and type IIb if MYH4 > 80%. The same muscle fiber types were combined using the Janus pipetting robotic system (Perkin Elmer).

### Tissue lysis and protein extraction

Whole skeletal muscles, as well as the liver, heart, and brain tissues, were snap-frozen in liquid nitrogen, deep-frozen, ground using a mortar and pestle, resuspended in 4% SDS in PBS, and then homogenized by heating for 5 min at 95 °C and subsequent sonication. Lysates were cleared by centrifugation for 10 min at 16,000 *g*, and protein concentrations were determined using the Pierce 660 nm Protein Assay (Thermo Scientific).

### Pulsed SILAC labeling in mice

Male adult wild-type mice (C57BL/J6) were fed a ^13^C_6_-lysine (Lys6)-containing mouse diet (Silantes) for 14 days to monitor newly synthesized proteins by comparing the incorporation of Lys-6 with the naturally occurring Lys-0 [19]. Mice were sacrificed at the end of day 14, and muscles were prepared for the ProFiT assay, as described above.

### Immunohistochemistry

Muscle cryosections (10 μm) were prepared after snap-freezing whole muscles in liquid nitrogen-cooled isopentane. Sections were fixed in − 20 °C cold acetone washed with Tris-buffered saline (TBS) and blocked for 1 h with 2% BSA, 5% horse serum, and 0.1% Triton X-100 in TBS. Sections were incubated with the primary antibody at 4 °C overnight, washed five times with TBS, incubated for 1 h with the secondary antibody, and then washed five times with TBS. Cryosections were mounted, or the previous steps were repeated (from blocking onwards) to immunohistochemically label other proteins. Microscopic pictures were captured on a DMi8A inverse microscope or a TCS SP8 gSTED 3X confocal microscope (both Leica). The following antibodies were used for staining: MYH2 (Developmental Studies Hybridoma Bank [DSHB], SC-71), MYH4 (DSHB, BF-F3), MYH7 (DSHB, BA-F8), TMEM65 (Sigma, HPA025020), TOM20 (abcam, ab56783), NPM1 (Santa Cruz Biotechnology, sc-56622), DES (Sigma, D1033), TPM1 (abcam, ab55915), TTN (kindly provided by Mathias Gautel, King’s College), CPS1 (abcam, ab45956), and Alexa Fluor secondary antibodies (Invitrogen).

### In-gel protein digestion

Proteomic samples of soleus type I and IIa fibers and type IIb fibers of the EDL, GAST, and TA were processed using a modified version of the in-gel digestion protocol by [20]. Briefly, ProFiT fiber pools and whole muscle lysates were precipitated by incubation for 4 h in four volumes (v:v) of ice-cold acetone. Protein pellets were extracted by centrifugation at 16,000 *g* for 10 min, dissolved in Bolt LDS sample buffer (Invitrogen), and then reduced and alkylated by incubation with 10 mM DTT and 55 mM IAA for 30 min at room temperature, respectively. Proteins were separated using Bolt 4–12% Bis-Tris Plus gels (Invitrogen), Coomassie-stained, and each lane was cut into eight slices. Each slice was chopped into 1 mm cubes, destained with 50% ACN, and dehydrated with 100% ACN. Samples were incubated at 37 °C overnight with 12 ng/μL Lys-C and trypsin (1:10-ratio), and the digestion was stopped by acidifying the samples with trifluoroacetic acid (TFA). Tryptic peptides were extracted with increasing concentrations of ACN, and organic compounds were evaporated using a SpeedVac concentrator Plus (Eppendorf). Acidified samples were desalted using Stop and Go extraction tips (StageTipping, [21]). We used in-house made StageTips filled with styrenedivinylbenzene- reverse phase sulfonate (SDB-RPS) material. StageTips were activated with 100% methanol, washed with 0.1% FA in 80% ACN, and equilibrated twice with 0.1% FA. Tips were centrifuged after each step to remove the liquids. The acidified samples were loaded onto the StageTips, washed once with 0.1% FA, twice with 0.1% FA in 80% ACN, and finally eluted with 1% ammonium hydroxide in 60% ACN. The samples were ready for LC-MS analysis after vacuum-drying in a SpeedVac concentrator and resuspension in 5% FA in 2% ACN.

### In-solution protein digestion

Lys-6-labeled samples were tryptically digested as described in [22]. In short, proteins were extracted as described previously, dissolved in urea buffer (6 M urea, 2 M thiourea in Tris buffer), reduced with 10 mM DTT (30 min, RT) and alkylated with 55 mM IAA (20 min in the dark, RT) and pre-digested with Lys-C for 1 h. Then, the urea concentration was reduced to < 2 M by adding ammonium bicarbonate buffer, and the samples were digested with Lys-C overnight at room temperature. The digestion was stopped by the addition of TFA, and the samples were desalted by StageTipping as described above.

### Phosphopeptide enrichment

Phosphopeptide enrichment requires high protein input amounts to ensure sufficient enrichment of phosphorylated peptides. We, therefore, collected from five wild-type mice two 96-well plates of soleus muscle fibers per animal. We pooled same fiber types of all animals into single samples for which we then performed tryptic digestion. Only for the phosphopeptide enrichment, we split the samples into three replicates which were, from then on, handled separately. Phosphopeptide enrichment was performed using a modified version of the protocol described by [23]. In brief, single fibers were lysed by incubation in GuHCl lysis buffer (6 M guanidine hydrochloride in 100 mM Tris-HCl pH 8.5, 5 mM tris (2-carboxyethyl) phosphine (TCEP), 10 mM 2-chloroacetamide CAA) for 10 min at 95 °C followed by sonication. Subsequently, fibers were characterized by the ProFiT approach and pooled according to the determined fiber type. Protein concentrations were determined as described previously. Proteins were digested by adding Lys-C for 2 h; 50 mM ammonium bicarbonate (ABC) buffer was added to dilute GuHCl below 2 M and finally, trypsin was added and incubated overnight at room temperature. The digestion was stopped by adding formic acid, and the peptides were desalted by solid-phase extraction (SPE) using OASIS HLB 1 cc 10 mg (Waters) columns. Columns were activated with 100% ACN and equilibrated with 0.1% FA. Peptides were loaded onto the columns, washed twice with 0.1% FA, eluted with 50% ACN containing 0.1% FA, and afterward, peptides were completely evaporated. TiO_2_ beads were incubated for 20 min in equilibration buffer (20 mg/mL dihydroxybenzoic acid [DHB] in 80% ACN, 6% TFA) on a rotating wheel and washed with elution buffer A (5% ammonium hydroxide), elution buffer B (10% ammonium hydroxide, 25% ACN), and twice with 70% ACN; then, the beads were incubated with equilibration buffer for 20 min. Dried peptides were resuspended in binding buffer (80% ACN, 12% TFA), and TiO_2_ beads were added (1:5 peptide-to-bead ratio), rotated for 15 min, pelleted by centrifugation, and the supernatant was transferred into a new reaction tube and the washing step was repeated. The bead-phosphopeptide complexes were transferred onto a previously prepared C8-StageTip and alternately washed and centrifuged with buffer A (10% ACN, 6% TFA), buffer B (40% ACN, 6% TFA), and buffer C (60% ACN, 6% TFA). Phosphopeptides were extracted by two consecutive elution steps with first, elution buffer A, and second, elution buffer B. Samples were evaporated until ~ 5 μL of liquid remained and finally desalted by StageTipping.

### LC-MS/MS analysis

In-gel digested proteomic samples were measured in data-dependent mode (DDA) using an Easy-nLC 1000 coupled to a Q Exactive Plus instrument (Thermo Scientific) via a nanoelectrospray ionization source. Peptides were separated based on hydrophobicity using a binary buffer system of buffer A (0.1% FA) and buffer B (80% ACN, 0.1% FA) on a 50-cm in-house-packed analytical column filled with 1.9 μm C18-AQ Reprosil-Pur beads (Dr. Maisch). In a 150 min chromatographic gradient, the amount of buffer B was linearly increased from 5 to 29% over 125 min, followed by a steeper linear increase of buffer B over 10 min to 95%. After washing with 95% buffer B for 5 min, the system was equilibrated by decreasing buffer B to 5% for 10 min. Full MS spectra (300–1750 m/z*)* were recorded at a resolution (R) of 70,000, maximum injection time (max. IT) of 20 ms, and AGC target of 3e6. The ten most abundant ion peptides in each full MS scan were selected for HCD fragmentation at nominal collisional energy (NCE) of 25. MS2 spectra were recorded at *R* = 17,500, max. IT of 60 ms, and an AGC target of 5e5. In-solution digested samples and phosphopeptides were analyzed using an Easy-nLC 1200 coupled by a nanoelectrospray ionization source to a Q Exactive HF-X. Pulsed SILAC samples were separated using a 90 min gradient with a linear increase of buffer B from 4 to 23% over 65 min, then increased to 55% over 13 min, and finally increased to 95% over 3 min followed by a total of 9 min washing with 95% buffer B. Full scan resolution was 60,000 with 20 ms max. IT and an AGC target of 3e6. The top 22 peptide ions were selected for HCD fragmentation (NCE = 28) and measured at *R* = 15,000, max. IT = 22 ms and AGC = 1e5. Phosphopeptide samples were analyzed using a 60 min chromatographic gradient (4–31% buffer B in 41 min, 31–58% buffer B in 9 min, 10 min washing in 95% buffer B). Full MS spectra were acquired at *R* = 60,000, max. IT = 20 ms and AGC = 3e6. The top 10 peptides were selected for HCD fragmentation (NCE = 28) and measured at *R* = 45,000, max. IT = 89 ms and AGC = 5e5.

### Data processing and analysis

DDA data were analyzed using MaxQuant (1.5.3.8, [16]). Peptides were matched against the mouse Uniprot database using the Andromeda scoring algorithm [17]. Multiplicity was set to one for proteomic and phosphoproteomic analysis; multiplicity was set to two with Lys-6 as the labeled amino acid for pulsed SILAC samples. Trypsin/P was chosen as the digesting protein for proteomic and phosphoproteomic analysis, and Lys-C/P was selected for pulsed SILAC samples, with a maximum of two missed cleavages for all samples. Carbamidomethylation of cysteine was set as a fixed modification, oxidation of methionine, and acetylation of protein N termini as variable modifications. Phospho (STY) was set as an additional variable modification for phosphopeptide analysis. Non-labeled samples were quantified using label-free quantification with the LFQ min. ratio count set to one. Peptide identification was set to a minimum of seven amino acids and an FDR < 0.01 and unique + razor peptides were used for quantification. The match between runs function was used.

Statistical analysis was performed in Perseus [24] and visualized using InstantClue [25]. Missing values were imputed by randomly picking values from a 1.8 standard deviation-downshifted normal distribution. Principal component analyses (PCA) were performed to calculate differences between sample groups. Student’s *t* tests were used for statistical testing, and 1D annotation enrichments [26] were used for the determination of enriched GO terms. Type IIb proteomes were analyzed by ANOVA multiple-sample testing; proteins identified as differentially regulated were hierarchically clustered using Euclidean distance. Fisher’s exact test was performed for every cluster to identify significantly enriched GO terms. Pulsed SILAC samples were statistically compared using a one-sample *t* test. Absolute protein copy numbers were calculated by using the proteomic ruler approach [27] and via the Proteomics Dynamic Range Standard kit (UPS2, Sigma). The proteomic ruler plugin (v.0.1.6) for Perseus was used to calculate copy numbers. UPS2 proteins were mixed with the muscle fiber lysates with a ratio of 1:100. MaxQuant protein search was performed with a FASTA database containing the human UPS2 proteins. UPS2 intensities were correlated to the known UPS2 protein concentration to calculate a linear regression which enabled the calculation of absolute protein copy numbers in single muscle fibers. Since we counted the average number of nuclei per fiber, we adjusted the absolute protein copy numbers to protein copy number per nucleus.

## Results

### Distinct protein patterns in intact skeletal muscle tissue and separated muscle fibers

**Fig. 1:**
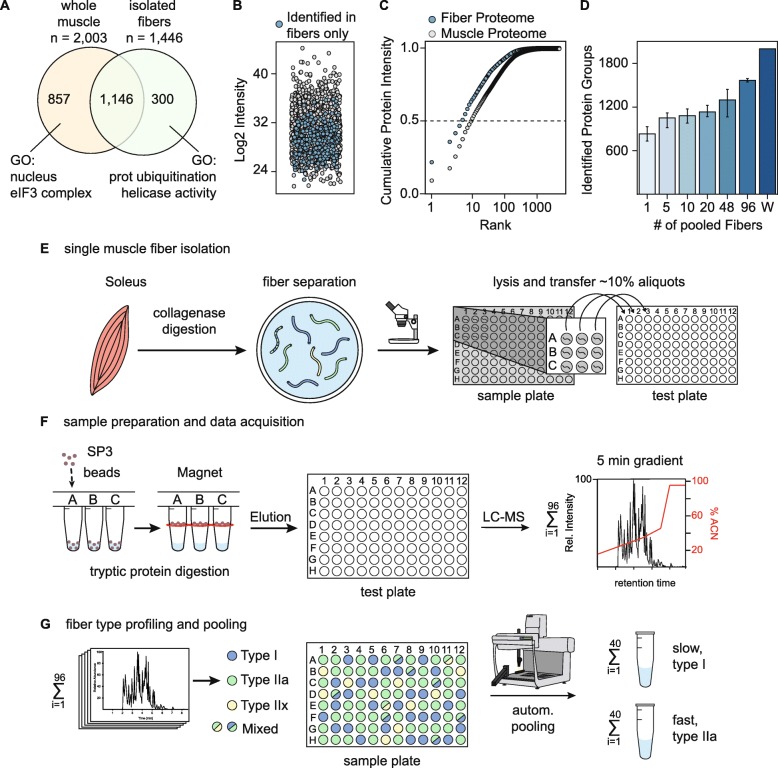
Distinct protein profiles between intact skeletal muscle tissue and separated fibers. **a** Venn diagram of protein groups identified in whole muscle lysates and pooled soleus muscle fibers (*n* = 48). **b** Jitter plot of skeletal muscle and fiber protein intensities (*n* = 48). Gray dots represent proteins identified in both skeletal muscle and fiber proteomes and blue labeled dots illustrate fiber-specific proteins. **c** Cumulative ranking of protein intensities for skeletal muscle (gray) and isolated fibers (blue; *n* = 48). **d** Number of proteins identified in 1 to 96 isolated muscle fibers from the soleus; W, whole skeletal muscle. **e**–**g** Overview of the experimental ProFiT workflow. **e** Isolation of single muscle fibers by collagenase digestion and manual fiber separation. **f** Sample preparation via SP3 beads and mass spectrometric analysis using short LC-MS gradients. **g** Data analysis and pooling of fibers with the same myosin heavy chain (MyHC) protein content by an automated liquid handling station

To provide an initial overview of the protein differences between skeletal muscle tissue and separated fibers, we extracted proteins from intact soleus skeletal muscles and a pool of ~ 50 isolated muscle fibers. All samples were in-solution digested and desalted peptides were analyzed by liquid chromatography–mass spectrometry (LC-MS). Quantitative MS analysis revealed 2003 protein groups in the intact muscle and 1446 protein groups in pure fibers **(**Fig. 1a, Table S1). To highlight the biological function of the proteins exclusively detected in intact muscles compared to single fibers, we applied gene ontology (GO) term enrichment using the GOrilla web tool [28]. In particular, the analysis showed an overrepresentation of the GO term “nucleus” in intact muscles suggesting that nuclei of non-muscular cells may contribute to myonuclear proteins [29]. Conversely, GO term analysis of 300 proteins that were exclusively detected in isolated fibers showed that protein ubiquitination and helicase activity were overrepresented. A jitter plot illustrates the low signal intensity of proteins found exclusively in muscle fibers compared to whole muscle extracts (Fig. 1b, blue circles). Protein ranking to assess the dynamic range of both samples showed that isolated myofibers have a higher dynamic range than whole muscles (Fig. 1c). Spike-in of a proteomics dynamic range standard (UPS2) allowed the calculation of protein copy numbers per fiber and substantiates the high dynamic range of these cells compared to undifferentiated cells [27, 30] (Suppl. Figure 1A, B). A comparison of fiber pools containing 1 to 96 fibers showed that an analysis of 20–50 fibers resulted in similar protein identification rates as ~ 100 muscle fibers (Fig. 1d).

### Single skeletal muscle fiber profiling using short capillary LC-MS gradients

Next, we isolated single muscle fibers from the soleus muscle of wild-type C57BL/6 J mice by collagenase digestion. Manually separated fibers were transferred to 96-well plates, dissolved in 20 μL lysis buffer, and sonicated to disrupt the fibers and extract proteins (Fig. 1e). From each sample, an aliquot of 2 μL (10%) was transferred to a test plate and subjected to a modified version of the single-pot solid-phase-enhanced sample preparation (SP3) method for tryptic protein digestion [15]. The digestion was performed using a 96-well microplate washer equipped with a magnetic plate (Fig. 1f). Testing short LC-MS/MS gradients on a linear ion trap Orbitrap and quadrupole Orbitrap instrument revealed ~ 180 peptides, which corresponded to 40 proteins per fiber after analysis of the RAW data with MaxQuant (Table S2; Suppl. Figure 1C, D). MyHC isoforms were among the most abundant proteins; on average, we observed 10 unique MyHC peptides per sample (Suppl. Figure 1E, Table S2). To reduce the measurement time for the MyHC profiling, we used a capillary LC system which enabled us to shorten the LC-MS gradient to 4 min with a reduced loading time delay, and a comparison between the instrumental setups revealed similar intensities for the detected MyHC peptides (Suppl. Figure 1F, G). This instrumental setup enabled us to analyze a 96-well plate within 8 h.

To test for carryover of MyHC peptides between samples, we recorded several blank runs alternated with single fiber samples and observed virtually no MyHC peptides in blank runs (Suppl. Figure 1H). Single muscle fibers were classified based on the highest relative abundance of a specific MyHC isoform relative to the overall abundance of all four fiber type-specific MyHC isoforms (MYH7, MYH2, MYH4, MYH1) per muscle fiber. We only selected unique MyHC peptides that represent one specific MyHC isoform to avoid incorrect peptide assignment; a representative selection of the most abundant unique MyHC peptides is listed in Suppl. Figure 1I-J; Table S2. As described in previous studies [14, 18, 31], fibers that expressed more than 60% (MYH1, MYH2) or 80% (MYH4, MYH7) of a specific MyHC isoform were pooled into the corresponding MyHC groups using an automated liquid handling system (Fig. 1g).

**Fig. 2:**
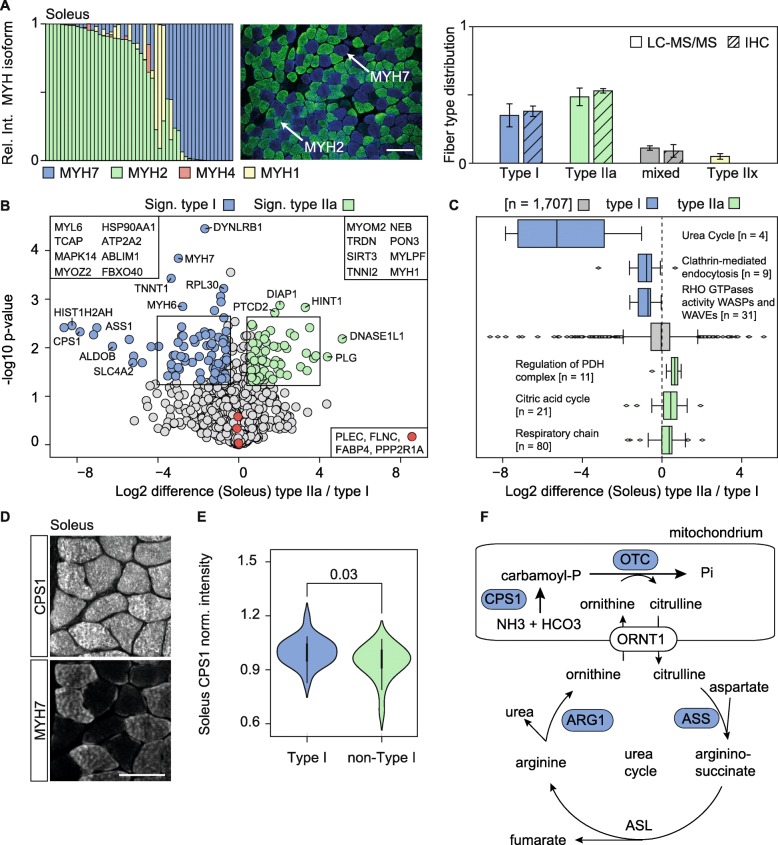
Proteomic profiling of slow and fast soleus muscle fibers reveals novel fiber type-specific marker proteins. **a** ProFiT analysis of isolated single fibers from the soleus (*n* = 4). Each bar represents the relative abundance of MYH isoforms in one fiber in relation to the total sum of intensities for MYH1, MYH2, MYH4, and MYH7. Immunostaining with MYH7 (slow type I, blue) and MYH2 (fast type IIa, green) antibodies. **b** Volcano plot comparing protein fold-changes (log_2_-scale) in soleus type I and type IIa fibers. Colored circles represent differentially regulated proteins (difference > 1.5-fold, *p* value < 0.05). **c** 1D annotation enrichment showing specific gene ontology terms enriched in soleus type I and type IIa fibers. **d** Co-immunostainings of carbamoyl-phosphate synthetase 1(CPS1) and myosin-7 (MYH7) on soleus cryosections (scale bar = 50 μm). **e** Quantitative analysis of the CPS1 fluorescent signal in type I (MYH7) positive and negative myofibers using violin plots. Significances were calculated by two-sided *t* tests (*n* = 42). **f** Graphical visualization of the urea cycle. Proteins involved in this pathway that were significantly enriched in soleus type I fibers are highlighted in blue

To confirm whether the fast-track MyHC profiling resulted in the same fiber type distribution as MyHC immunostaining protocols, we screened ~ 780 isolated fibers from the soleus from four independent animals (*n* = 4) and measured all fibers using a short LC-MS gradient (Fig. 2a). The comparison demonstrated a similar fiber type distribution between ProFit and immunostaining. Furthermore, ProFiT enabled the detection of 33 type IIx fibers (MYH1) which cannot be detected by immunostaining [32, 33].

### Proteomic analysis reveals distinct protein expression patterns between different muscle fiber types

To analyze the proteomes of fibers derived from different muscles, we screened 400 fibers from the soleus, EDL, gastrocnemius (GAST), and tibialis anterior (TA) muscles. We then created individual pools of ~ 30 type I soleus fibers, type IIa soleus fibers, and IIb fibers from the EDL, GAST, and TA. Samples were in-gel digested, subjected to LC-MS, and analyzed by MaxQuant using the match between runs option in combination with a peptide library generated from intact muscles to increase the number of protein hits [14]. In total, we identified 27,899 peptide sequences and 2453 different protein groups in soleus type I and type IIa fibers, with a false discovery rate (FDR) below 1% at the protein and peptide level (Table S3). On average, ~ 1700 proteins were quantified per isolated fiber type. We performed principal component analysis (PCA) to determine whether the MS analysis of type I and IIa fibers revealed distinct protein profiles. Clear separation of both fiber types was observed in the first two components (Suppl. Figure 2A). The main differences distinguishing type I and IIa fibers are mainly slow and fast isoforms of known sarcomeric proteins, such as MyHC, troponin, tropomyosin, and calcium pump isoforms (Suppl. Figure 2B). Next, we performed a two-sided *t* test using the log_2_ +/− 0.58 ratio and the − log_10_*p* value > 1.3 as a cutoff to identify significantly regulated proteins between type I and IIa fibers (Fig. 2b). In total, 180 proteins were differentially expressed, and a 1D annotation enrichment revealed proteins associated with the GO terms urea cycle, RHO GTPases activity, and clathrin-mediated endocytosis to be enriched in soleus type I fibers (Fig. 2c, f). Contrasting, type IIa fibers from the same muscle group showed increased levels of mitochondrial proteins, reflecting the presence of higher numbers of mitochondria in fast oxidative type IIa fibers [33] (Fig. 2c).

The abundance of the transmembrane protein TMEM65 was increased in type IIa fibers (ratio 2.0; *p* value 0.09). TMEM65 has been described as an intercalated disc protein that interacts with connexin-43 in heart tissue [34]. Another study localized TMEM65 to the inner mitochondrial membrane [35]. Here, we confirmed the mitochondrial localization of TMEM65 by co-immunostaining soleus cryosections with TOM20 (Suppl. Figure 2e). In addition, MYH2 and MYH7 co-immunostaining of the soleus and EDL confirmed that TMEM65 is mainly expressed in type IIa fibers (Suppl. Figure 2F-I), which have a higher density of mitochondria than type I and type IIb fibers. Several mitochondrial proteins, e.g., CPS1 and OTC of the urea cycle (Fig. 2f) showed increased levels in soleus type I fibers. We confirmed the increased levels of the mitochondrial protein CPS1 in soleus type I fibers by co-immunofluorescent stainings of soleus cryosections (Fig. 2d–e).

**Fig. 3:**
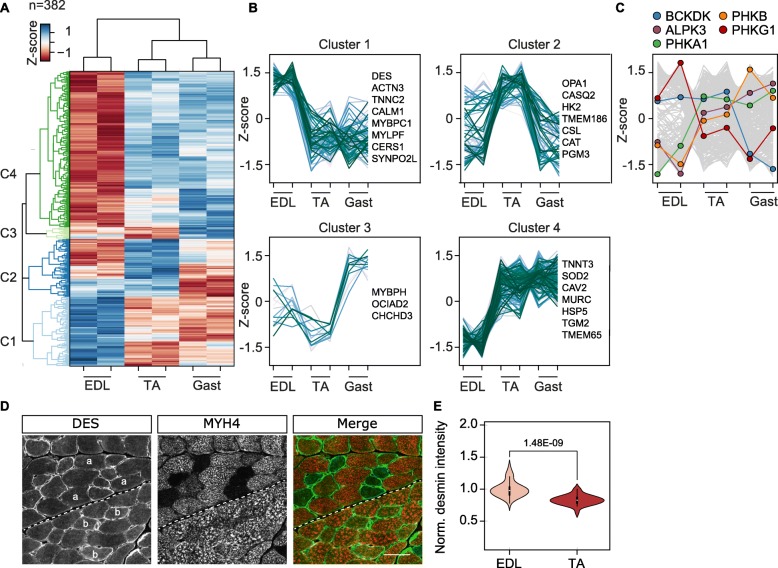
Isolated type IIb muscle fibers from EDL, TA, and Gast muscles exhibit distinct protein profiles. **a** Hierarchical clustering of significantly regulated proteins (ANOVA *p* value < 0.05; **Table S4**) between type IIb fibers (expressing the MYH4 isoform) from the EDL, TA, and Gast muscles. **b** Z-score distribution of proteins in clusters 1–4. Selected proteins are labeled next to the graphs. **c** Kinases in type IIb fibers with different expression profiles in the EDL, TA, and GAST; branched-chain ketoacid dehydrogenase kinase (BCKDK), phosphorylase b kinase regulatory subunit beta (PHKB), phosphorylase b kinase γ catalytic chain, skeletal muscle isoform (PHKG1), phosphorylase b kinase regulatory subunit α, skeletal muscle isoform (PHKA1), and α-protein kinase 3 (ALPK3). **d** Co-immunostaining of desmin (DES) and myosin-4 (MYH4) in muscle cryosections depicting the TA (above dashed line) and EDL muscle (below dashed line). Type IIb fibers are marked by “a” (TA) or “b” (EDL; scale bar = 50 μm). **e** Quantitative analysis of the desmin fluorescence signal in type IIb (MYH4) positive myofibers in the TA and EDL muscles. Significances were tested by two-sided *t* tests (*n* = 74)

Next, to provide a statistical overview of the data, we performed a multiple-sample test (ANOVA) to identify significantly regulated proteins in type IIb fibers from the EDL, GAST, and TA (Table S4). We identified in total 2411 proteins, including 382 significantly regulated proteins in type IIb fibers of the three muscle groups tested (ANOVA significant − log_10_*p* value > 1.3). PCA analysis showed a clear separation of IIb fibers between the three muscle groups (suppl. Figure 2C-D). Furthermore, *Z* score normalization of all significantly regulated proteins followed by Euclidean hierarchical clustering revealed a closer similarity between type IIb fibers from the TA and GAST than type IIb fibers from the EDL (Fig. 3a). A Fisher’s exact test of all proteins in cluster 1–4 revealed enrichment of GO terms related to fiber contractility (cluster 1) and mitochondrial proteins (clusters 2 and 4; Table S4, Fig. 3b).

For example, the actin-binding protein α-actinin-3 showed the highest protein expression of type IIb fibers in the EDL, supporting previous studies that high α-actinin-3 levels are required to maintain the fast contractile properties of the EDL muscle group [36, 37]. The isoform of tropomyosin TPM1 also showed an increased protein expression in EDL type IIb fibers, and we substantiated this by immunostaining EDL and TA cryosections (Suppl. Figure 4A-B). Interestingly, we also identified a significantly increased protein expression of desmin in type IIb fibers of EDL compared to IIb fibers of TA **(**Fig. 3d–e). As a control, we used the giant protein titin, which is equally expressed between all IIb fibers of TA and EDL (Suppl. Figure 4C-D).

In addition, cluster 2 is comprised of ten proteins associated with the GO term aging (GO: 0007568), including the proteins catalase and dystrophin. We also observed increased levels of the alpha and beta subunits of the phosphorylase kinase (PHKA1, PHKB) from the EDL < TA < GAST. PHK is responsible for catalyzing the first step of glycogen breakdown and may reflect a shift from the inactive T-state towards the active R-state of glycogen phosphorylase (GP). Conversely, the opposite trend was observed for the gamma subunit of PHK, with enhanced levels in the EDL compared to the TA and GAST.

Overall, our fiber type-specific quantitative proteome analysis confirmed metabolic adaptions in oxidative and glycolytic fibers and highlights significant differences between the proteomes of type IIb fibers from different muscle groups.

### Lys-6 incorporation rates reveal differential protein synthesis between slow and fast fibers

**Fig. 4:**
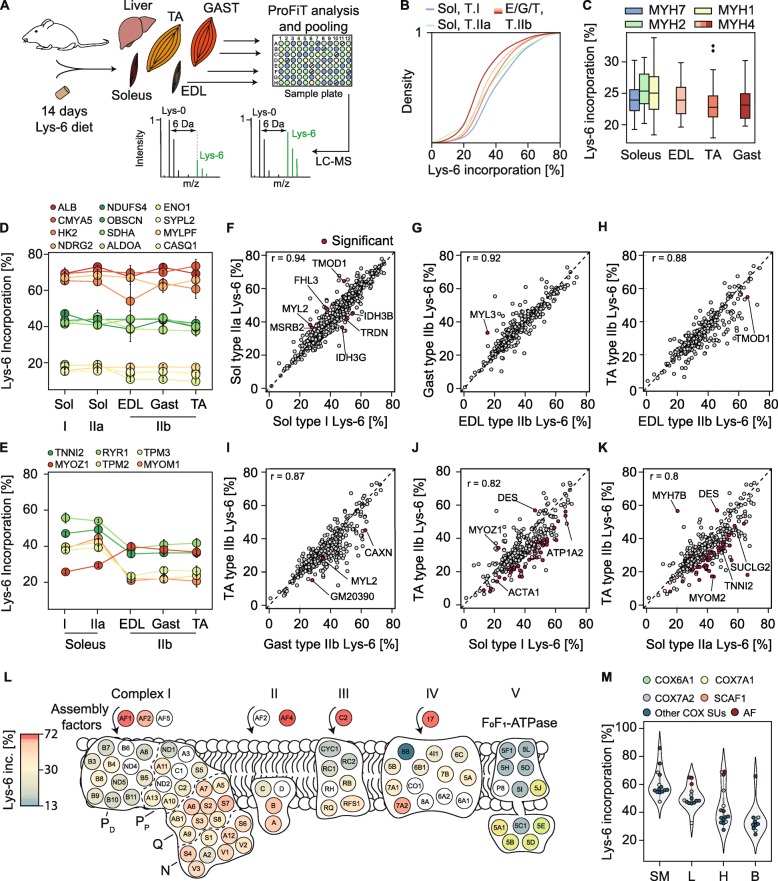
In vivo pulsed SILAC reveals enhanced Lys-6 labeling for assembly factors and *OXPHOS* complex subunits. **a** Schematic overview of Lys-6 administration for 14 days followed by the ProFit assay. Experiments were conducted in biological quadruplicates (*n* = 4). Theoretical MS SILAC pairs indicate proteins with slow and fast turnover. **b** Density plot of Lys-6 incorporation into different fiber types from the soleus (Sol), EDL (E), Gast (G) and tibialis anterior (T). Type I soleus fibers (depicted in blue) exhibited the highest Lys-6 incorporation rate. **c** Boxplot analysis of Lys-6 incorporation into different MYH isoforms in the soleus, EDL, TA, and Gast muscle groups. **d** Selected proteins with high, intermediate and low Lys-6 incorporation into newly synthesized proteins. **e** Selected proteins with different Lys-6 incorporation between slow (soleus) and fast muscle fiber types (EDL, Gast, TA). **f**–**k** Scatter plots for different muscle fiber types from distinct muscle groups. Red dots represent proteins with significantly different Lys-6 (H)-to-Lys-0 (L) ratios (one-sample *t* test; *p* value < 0.05 and abs. difference > 1.5). **l** Map of OXPHOS proteins and assembly factors and their Lys-6 incorporation rates. Complex I modules are indicated by P_D_, P_P_; Q and N. **m** Lys-6 incorporation rates for complex IV subunits and complex IV assembly factors (AF) in the skeletal muscle (SM), liver (L), heart (H), and brain (B) tissue in biological triplicates (*n* = 3)

Previous experiments based on the uptake of radioactive tracers showed that slower muscles have a higher turnover rate than more glycolytic and faster muscles [9]. However, the extent to which fiber types and individual proteins contribute to muscle turnover rates remained unclear. To assess the synthesis of individual proteins, we fed mice a ^13^C_6_-lysine (Lys-6)-containing diet for 14 days to label newly synthesized proteins (Fig. 4a). Muscle fibers from the soleus, EDL, TA, and GAST were isolated, and the ProFiT approach was used to classify fiber types. After pooling fibers with the same MyHC isoform expression, we were able to quantify ~ 1720 proteins. Lys-6 labeling ranged from ~ 10 to ~ 80% after 2 weeks of Lys-6 administration (Table S5, Suppl. Figure 5A). A density plot of incorporation rates confirmed higher Lys-6 labeling of soleus slow type I fibers compared to type IIb fibers from the EDL, GAST, and TA muscles (Table S5, Fig. 4b). This shift was also reflected by higher Lys-6 incorporation rates for MyHC isoforms in the soleus compared to the fast MYH4 type IIb isoform in the EDL, TA, and GAST (Table S5, Fig. 4c).

The liver, the major source of the blood proteins, exhibited clearly enhanced Lys-6 incorporation rates compared to other non-proliferating tissues such as the muscle and heart (Suppl. Figure 5B) [19]. For example, serum albumin (ALB) had Lys-6 incorporation rates of ~ 70% in the liver and isolated muscle fibers (Fig. 4d, suppl. Figure 5A-B). Based on these identical incorporation rates, we conclude that the accumulation of newly synthesized albumin in muscle fibers originates from the uptake of albumin from the blood liver system; albumin is unlikely to be synthesized in skeletal muscle tissues. However, cell-type-specific labeling of newly synthesized proteins should be performed to identify the location of albumin synthesis [38].

The highest Lys-6 incorporation rate for a non-serum protein in soleus type I and IIa fibers was observed for coiled-coil-helix-coiled-coil-helix domain-containing protein 2 (CHCHD2; Lys-6 labeling ~ 75% +/− 2). This protein is a transcriptional activator of COX4I2 expression that modulates COX activity in response to altered energy conditions [39]. The high CHCHD2 Lys-6 incorporation rate in soleus type I and IIa fibers may reflect the high demand for mitochondrial OXPHOS complexes in muscle fibers. Interestingly, several sarcomeric proteins, including the nebulin-related anchoring protein (NRAP), leiomodin-3 (LMOD3), and the small muscular protein (SMPX), were exclusively detected in type I and IIa fibers; moreover, these sarcomeric proteins had extremely high Lys-6 incorporation rates of over 70% after administration of Lys-6 for 2 weeks (Table S5). Representative SILAC MS spectra of myomesin-2 (MYOM2), isocitrate dehydrogenase 3G (IDH3G), and titin are shown in Suppl. Figure 5C and illustrate the different Lys-6 incorporation rates between type I and IIa fibers of the soleus. Proteins with similar Lys-6 incorporation rates between the tested fiber types are illustrated in Fig. 4d. For example, the stress-responsive protein tumor suppressor N-myc downstream-regulated gene 2 (*NDRG2*) is expressed at high levels in striated muscle and exhibited a uniform incorporation rate of 66% +/− 7% between all muscle fibers and muscle groups after 14 days Lys-6 labeling (Fig. 4d). Conversely, histones, enolase 1 (ENO1) and calsequestrin-1 (CASQ1) showed constant low Lys-6 labeling rates ranging from 9 to 20%, indicating increased half-lives for these proteins compared to other skeletal muscle proteins. Moreover, we also identified several proteins with distinct protein turnover rates between the three fiber types (Fig. 4e). For example, myozenin-1 (also known as calsarcin-2; FATZ) had a higher Lys-6 incorporation rate in type IIb fibers than the tested soleus type I and IIa fibers. Conversely, TNNI2 and TPM3 had a higher Lys-6 incorporation rate in slow type I and IIa fibers than IIb fibers.

We next plotted Lys-6 incorporation rates between type I, IIa, and IIb fibers (Fig. 4f–k) and observed a high Pearson correlation (*r* = 0.94) for the comparisons of type I and IIa fibers within the same muscle groups, indicating similar turnover rates for most of the identified proteins. It is important to note that the mitochondrial proteins IDH3B and IDH3G, which are expressed at higher levels in type IIa fibers (Table S3), showed significantly increased Lys-6 labeling in slow type I fibers than type IIa fibers (Fig. 4f, 1-sample *t* test of Lys-6-to-Lys-0 ratios). Lower Pearson correlations (*r* = 0.82 and *r* = 0.80) were obtained for the comparison of type IIb fibers from the TA with type I and IIa fibers from the soleus (Fig. 4j, k).

A detailed map of the Lys-6 incorporation rates of OXPHOS subunits revealed a broad range of labeling, which was dependent on the localization of the subunits within distinct complexes (Fig. 4l). The highest Lys-6 incorporation rates were observed for respiratory chain complex assembly factors. MS detected 39 of the 45 subunits of complex I. Comparison of Lys-6 labeling rates in complex I submodules (P_D_-, P_P_-, Q-, and N-module) demonstrated significantly higher Lys-6 labeling of proteins localized in the N- and Q-modules at the matrix site compared to the members of the P-modules (Fig. 4l, complex I, Table S5). The matrix site of complex I is the major entry point for electrons into the respiratory chain, thus the close proximity of these proteins to reactive oxygen species (ROS) may result in oxidative damage to complex I subunits. Similarly, previous radioactive pulse-chase experiments showed an enhanced exchange of N- and Q-module members [40–42]. Hence, our pulsed SILAC approach reflects the dynamic synthesis of the subunits in the matrix arm of complex I in skeletal muscle fibers. Notably, compared to other N-module subunits, NDUFA2 had significantly lower Lys-6 incorporation (~ 30%). Since NDUFA2 is catalytically inactive and functions as an accessory subunit involved in complex I biogenesis, NDUFA2 may be less affected by ROS, resulting in a lower synthesis rate [43, 44]. It is important to note that all assembly factors of the mitochondrial respiratory chain detected—including NDUFAF1, NDUFAF2, SDHAF4, UQQC2, and COX17—had the highest Lys-6 incorporation rates of all OXPHOS proteins, reflecting the high turnover of these factors.

In addition, previous studies showed that the formation of the CIII-CIV super-complex is modulated by the assembly factor COX7A2L (also known as SCAF1). However, since SCAF1 was not detectable in our analysis of skeletal muscle tissue of C57BL/6 J mice, we observed a very high Lys-6 incorporation rate for the related cytochrome C oxidase COX7A2 (53% +/− 4%), similarly to the aforementioned assembly factors (Table S5, Fig. 4l).

To demonstrate that SCAF1 has a high Lys-6 incorporation rate, similar to other assembly factors, we examined Lys-6 incorporation in the heart, brain, and liver tissues. The Lys-6 incorporation rate of complex IV subunits decreased from skeletal muscle (SM) to the liver, heart, and brain. In the heart and liver tissues, the cytochrome C oxidase assembly factors COX17, COX19, and COX20 (Fig. 4m, *red circles)* had the highest Lys-6 incorporation rates, followed by SCAF1 (*light red circles*). Taken together, these findings suggest that COX7A2 (*purple circles***)** might also participate in complex assembly in skeletal muscle tissues.

### Phosphoproteomics reveals distinct kinase activities between muscle fibers

**Fig. 5:**
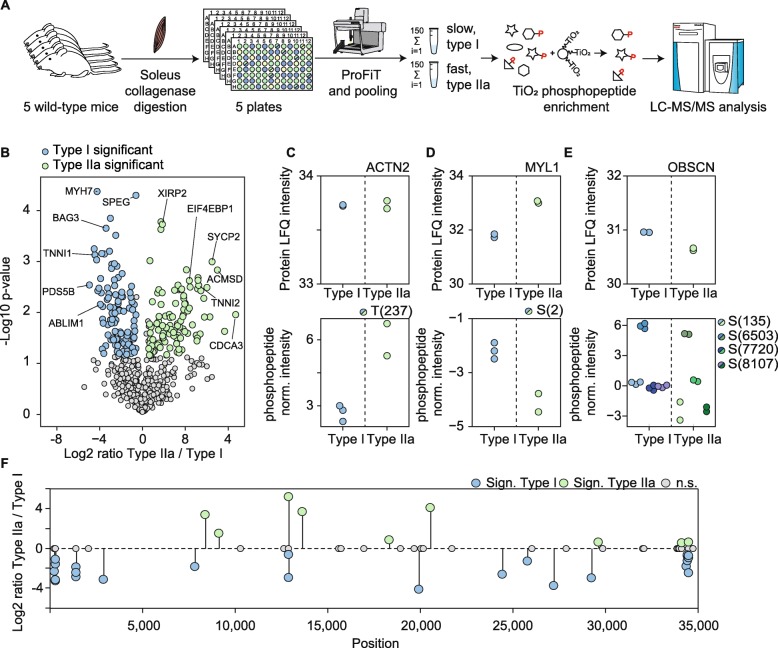
Phosphopeptide enrichment identifies different protein phosphorylation profiles in slow and fast muscle fiber types. **a** Overview of the experimental setup for phosphopeptide enrichment. Approx. 1000 fibers from separated soleus muscles (*n* = 5) were screened via the ProFiT assay. Fibers were pooled according to their phenotype and distributed to three technical replicates per fiber type. Phosphopeptide enrichment of soleus type I and IIa fibers was performed using titanium dioxide beads (TiO_2_). **b** Volcano plot comparing phosphopeptide fold-changes between type I and type IIa fibers from the soleus. Colored circles indicate significantly regulated phosphorylation sites (abs. log_2_ fold-change > 0.58 and *q* value < 0.05). **c**–**e** Selected protein LFQ intensities (upper panel) and normalized phosphopeptide intensities (lower panel) for significantly different phosphorylation sites on actinin-2 (ACTN2), myosin light chain 1 (MYL1), and obscurin (OBSCN). **f** Map of titin phosphopeptides. The color code indicates non-regulated and significantly regulated phosphorylation sites (abs. log_2_ fold-change > 0.58 and *q* value < 0.05)

To further elucidate the versatility of our approach, we conducted a fiber type-specific phosphoproteomics experiment based on label-free quantitation. To dissect the differences in protein phosphorylation in type I and IIa fibers from the soleus, we collected ten 96-well plates containing single muscle fibers from the soleus of five wild-type mice (*n* = 5) and conducted ProFiT screening for each plate. We then mixed ~ 300 fibers expressing the same MyHC isoforms, yielding 150 μg protein input per fiber type. Phosphopeptide enrichment was performed via titanium-dioxide beads (Fig. 5a), which enabled the identification of more than 900 phosphorylation sites covering 226 phosphoproteins (Table S6). Statistical analysis revealed 219 significantly regulated phosphorylation sites between soleus type I and IIa fibers (Fig. 5b, FDR significant + abs. diff > 1.5).

For instance, the Z-disk- and actin-binding protein α-actinin-2 was expressed at equal levels in type I and IIa fibers (Table S3, Fig. 5c) but showed increased phosphorylation at T237 in type IIa fibers (Fig. 5c). This phosphorylation site is located within the second calponin homology region; this domain is responsible for actin binding; thus, its phosphorylation may affect protein-protein interactions in muscle fibers. In addition, we identified 28 phosphorylation sites on MyHC isoforms.

Myosin molecules are tightly associated with different myosin light chains, modulating the stability of the myosin head, contributing to force production, and altering the association with the actomyosin network [45, 46]. The regulatory isoforms MYL2 and MLC2F are phosphorylated at S14 and S15, and these modifications are associated with force potentiation in fast skeletal muscles [47, 48]. We found equal levels of S14 and S15 phosphorylation in type I and IIa fibers, suggesting similar regulation of these light chains in soleus fibers.

The function of phosphorylation of the essential/alkaline myosin light chain 1/3, skeletal muscle isoform (gene: *Myl1*) is unclear. We observed significantly increased phosphorylation of serine 2 (S2) of the MLC3 isoform in type I fibers compared to type IIa fibers (Fig. 5d). Another sarcomeric protein, obscurin (OBSCN), is closely associated with the M-band, and the existence of a calmodulin-binding IQ motif and a Rho-guanine nucleotide exchange factor domain suggest that obscurin is involved in Ca2+/calmodulin and G-protein coupled signal transduction in the sarcomere [49]. Eighty-six phosphorylation sites have been identified in obscurin, though the functions of most of these sites are unknown. Six obscurin phosphorylation sites were present at similar levels in type I and IIa fibers, three sites (S135, S6503, S8107) were significantly increased in type I fibers and one phosphorylation site (S7220) was significantly increased in type IIa fibers (Fig. 5e).

We observed 89 phosphorylation sites in the giant protein titin, mainly localized at the N- and C-termini (Fig. 5f). Moreover, our quantitative analysis uncovered differential regulation of 35 titin phosphorylation sites between type I and IIa fibers (Fig. 5f). Titin has two main functions in muscle fibers: stabilizing the sarcomere structure and most importantly, as a molecular spring responsible for the relaxation of contracted sarcomeres [50]. Since we observed 24 phosphorylation sites with increased levels in type I localized in the N-terminus of titin or close to Ig-like domains of the I-band region, we suggest that these sites may affect the flexibility of titin in this region. These data demonstrate that muscle fibers with distinct MyHC protein expression exhibit distinct phosphoproteomes, even for fibers within the same muscle group.

## Discussion

Intact skeletal muscle tissue consists of a network of different cell types and extracellular structures. Therefore, analyses of whole muscle protein extracts always reflect a mixture of cell types, and it is difficult to unambiguously assign proteins to a specific cell population. In order to obtain a more focused view of the fiber proteome, previous reports analyzed individual fibers to identify physiological adaptions to the mitochondria or assess differences in the response to disease-related conditions. However, detailed proteomic and phosphoproteomic analysis of single muscle fibers is impeded by the low quantities of protein in single muscle fibers.

We used short LC-MS gradients to screen MyHC isoforms in a 96-well format, which allowed us to collect and pool sufficient numbers of fibers with the same MyHC expression and overcome the limited quantities of protein in single fibers for mass spectrometric (MS) analysis. It is important to note that the use of a capillary LC system with short 5-cm reverse phase columns and short LC gradients enables screening of ~ 300 fibers per day.

Comparing our ProFiT approach with immunostaining of fibers in tissue sections revealed similar fiber type distributions for both methods (Fig. 2a), and the MS analysis of MyHC isoforms enabled quantification of the MYH1 isoform (type IIx), which is difficult using antibody-based techniques. However, we are aware that our approach does not specify regional differences between fibers within larger muscle groups. Collagenase digestion of large muscles such as the GAST or quadriceps (QUAD) does not lead to completely uniform separation of all muscle fibers. Hence, smaller muscles such as the soleus, plantaris, FDB, TA, EDL, and extraocular muscles are better suited for MS-based fiber type screening. Alternatively, one could use manual disruption methods, without enzymatic digestion, to investigate specific areas of larger muscle bundles.

The results of our protein profiling of different fiber types are in agreement with earlier reports describing the distribution of slow and fast isoforms, the adaption to energy production and protein turnover.

Proteins of the transmembrane (TMEM) family are associated with various cell membranes and fulfill important physiological functions such as myoblast fusion (TMEM8c, also named myomaker) and the assembly of OXPHOS complex I (TMEM261) [51]. We observed equal distribution of the mitochondrial membrane protein TMEM261 between type I and IIa fibers, whereas TMEM65—another inner mitochondrial membrane protein—was upregulated in type IIa fibers compared to type I fibers (Table S3). Genetic ablation of *Tmem65* results in mitochondrial OXPHOS dysfunction, and human patients with *TMEM65* mutations develop severe encephalomyopathic mitochondrial disease [52]. Whether TMEM65 specifically modulates mitochondrial activity and respiration in type IIa fibers is unclear. A fiber type-specific proteomic study of *Tmem65-*deficient muscles could shed light on the function of this mitochondrial membrane protein. Systematic GO analysis revealed enrichment of proteins associated with vesicle transport and endocytosis in slow type I fibers compared to fast fibers (Fig. 2c). This clearly reflects the increased uptake and transport of metabolites, including glucose, fatty acids, and amino acids, in slow fibers compared to fast fibers [53]. Overall, our results demonstrate physiological differences between muscle fiber types, and our quantitative proteome analysis highlights several proteins with a previously unknown fiber type annotation.

Mitochondria are cellular compartments that clearly adapt to metabolic activities [18], and accordingly, each fiber type exhibits adaptations to the number, activity, and protein content of its mitochondria [54]. For example, direct comparison of the signal intensities of all mitochondrial proteins in soleus type I and IIa fibers revealed a slight shift in protein intensities towards IIa fibers, reflecting the higher density of mitochondria in this fiber type (Suppl. Figure 3, Table S3) [55].

The metabolites of the TCA cycle, a crucial energy generator, are closely associated with the urea cycle via the aspartate-argininosuccinate shunt (also known as the Krebs bicycle). Our fiber type-specific proteomics study revealed enhanced protein abundances of four urea cycle enzymes (CPS1, OTC, ARG1, and ASS) in type I fibers compared to IIa fibers (Fig. 2f). Fumarate produced by ASS and ASL is an intermediate of the TCA cycle, enters mitochondria, and is converted to oxaloacetate. Given the differential regulation of urea-cycle enzymes between fiber types, particularly in the context of the associated metabolites, it is also likely that the product molecules are differentially utilized for synthesis of creatinine, nitric oxide, and polyamines. Thus, the combination of ProFiT with metabolomics approaches could potentially provide a tool to quantify small molecules in different fiber types with distinct metabolic activities and that analysis of the amino acids arginine, ornithine, and citrulline could help to decipher fiber type-dependent metabolite fluxes.

The enzyme isocitrate dehydrogenase 2 (IDH2), a TCA cycle protein that plays a role to protect against ROS, converts isocitrate to α-ketoglutarate. This reaction produces NADPH, an important molecule involved in protection against ROS damage. Similar to previous single muscle fiber studies, we also observed increased levels of IDH2 in soleus type I fibers, whereas type IIa fibers of the soleus predominantly expressed IDH3 [18, 31]. Interestingly, the Lys-6 incorporation rates of all IDH isoforms were significantly higher in type I fibers than IIa fibers. It is tempting to speculate that increased ROS levels could lead to higher levels of protein damage, and increased Lys-6 labeling of IDH2 and IDH3 could reflect compensation for increased loss of these proteins in type I fibers. Analysis of mitochondrial OXPHOS complexes revealed similar Lys-6 incorporation rates (~ 32%) for most of the subunits of complex I–V across all fiber types. However, the subunits localized in the N- and Q-modules of complex I showed significantly higher Lys-6 incorporation rates than the P-module subunits localized within the inner mitochondrial membrane. Our results are consistent with turnover studies based on radiolabeling, demonstrating specific exchanges of subunits within intact complexes to prevent the accumulation of damaged N- and Q-module subunits, which are in direct contact with ROS [42].

All of the mitochondrial respiratory chain assembly factors detected, including NDUFAF1, NDUFAF2, SDHAF4, UQCC2, and COX17, showed high incorporation rates in skeletal muscle fibers, indicating high turnover of these assembly factors. Similarly, proteins involved in the formation of the CIII-CIV super-complex including the cytochrome c oxidases COX7A2L (SCAF1), COX7A2, and COX6A also had high Lys-6 incorporation rates. COX7A2 is replaced either by COX7A1 for CIV dimer formation or by SCAF1 for the assembly of supercomplexes containing CI, CIII, and CIV [56]. We assessed whether the Lys-6 incorporation rates of these proteins are equally high in other tissues and measured labeled liver, heart, brain and intact skeletal muscle tissue from the soleus to identify tissue-specific isoforms (Fig. 4m). We detected high Lys-6 labeling for several assembly factors, including COX17, COX19, COX20, SCAF1, and COX6A1/2 in the liver and heart tissue. This result confirmed the high synthesis rates of assembly factors. Hence, following Lys-6 incorporation rates under conditions that promote elevated assembly of the respirasome super-complex could help to elucidate the protein dynamics of individual respiratory complexes [57–59].

Since we were able to collect sufficient protein material to enable analyses of each fiber type, we wondered whether we could analyze the phosphoproteome of type I and IIa fibers from the soleus. We previously identified that the protein expression levels of several kinases and phosphatases vary between different fiber types, suggesting that protein phosphorylation levels may also vary [14, 60]. In agreement with this hypothesis, we observed several differential phosphorylation sites between the investigated fiber types (Fig. 5b). In particular, 35 of the 87 phosphorylation sites identified on the giant protein titin were differentially regulated. Since the functions of most of the reversible phosphorylation sites on titin are unknown, our fiber type-specific phosphopeptide mapping will contribute to exploration of how titin phosphorylation and dephosphorization modulates the contractility and elasticity of skeletal muscle fibers [61].

## Conclusion

Overall, this series of experiments demonstrate the ProFiT approach enabling rapid screening of hundreds of muscle fibers. It can be used to amplify the amounts of protein to achieve comprehensive proteomic and PTM analysis of specific fiber types. Furthermore, our results demonstrate a number of physiological differences between muscle fibers, and the quantitative proteomic analysis highlights several proteins with previously unknown fiber type annotations.

## Supplementary information

**Additional file 1 **: **Suppl. Figure 1: LC-MS gradient optimization and identification of unique peptides from different MYH isoforms. A)** Calculation of absolute copy numbers per nucleus by spike-in of the Proteomics Dynamic Range Standard (UPS2) indicates the high dynamic range of muscle fibers. The detection limit was estimated to be ~200 protein copies per muscle fiber nucleus. Conversely, the most abundant protein MYH1 had a copy number of 3x10^11^ copies per nucleus. The dashed red line indicates a local regression (locally weighted scatterplot smoothing). **B)** Absolute protein copy numbers calculated with the UPS2 kit or the Proteomic Ruler approach. **C-E)** Bar diagrams indicating identified **C)** peptides, **D**) proteins and **E)** unique MYH peptides identified from 23 single muscle fibers of the soleus muscle using a linear ion trap instrument. LC-MS gradients ranged from 10 min to 240 min. **F**) Muscle fiber type distribution between a linear ion trap Orbitrap (LTQ Orbitrap) coupled to an Easy nLC II and a quadrupole Orbitrap (QExactive HF-X) instrument coupled to a Dionex Ultimate 3000 UHPLC. Colored dots indicate the predominant MYH protein in the respective muscle fiber. **G**) Comparison of the top 6 MYH1, MYH2 and MYH7 peptide intensities identified on an Easy nLC II-LTQ Orbitrap setup and a CAP-LC-QExactive HF-X setting. **H**) Measurement of peptide carry-over after a ProFiT run using 10 min chromatographic gradients. No detectable carryover of peptides was observed between samples of single muscle fibers and blank runs. The measured intensities of blank runs were 2-3 orders of magnitude lower compared to the single muscle fiber samples and virtually no MYH peptides were detectable in blank runs. **I-J**) Top five most abundant unique MYH peptides from three different isoforms were identified using either **I)** the Easy nLC-II-LTQ Orbitrap or **J)** the CAP-LC-QExactive HF-X setting. The peptide position within each protein is illustrated in the upper panel. The exact position and retention times are listed in Table S2.

**Additional file 2 **: **Suppl. Figure 2: Systematic view of protein changes between slow and fast muscle fibers. A**) Principal component analysis (PCA) revealed a clear separation of type I and type IIa fibers from the soleus muscle and **B**) indicates the main components responsible for the separation (green for type IIa and blue for type I). **C**) Similarly, PCA clearly confirmed the separation of protein intensities for type IIb fibers from the EDL, TA, and Gast. **D**) Blue labeled circles represent the main components responsible for the separation. **E**) Overview of co-immunostaining of soleus cross-sections with antibodies that recognize TMEM65 and the mitochondrial outer membrane protein TOM20. **F**) Co-immunostaining of soleus cross-sections with TMEM65 and antibodies that bind specifically to slow MYH7 (type) and fast MYH2 (type IIa) isoforms. **G**) Quantitative analysis of TMEM65 fluorescence signal intensities in different fiber types in the soleus muscle. Significance was tested by two-sided t-tests (*n* = 297). **H**) EDL cross-sections immunostained with TMEM65, MYH2, and MYH4. **I**) Quantitative analysis of the TMEM65 fluorescence signal in EDL myofibers. Significances were tested by two-sided t-tests (*n* = 200)

**Additional file 3: **: **Suppl. Figureure 3: Overrepresentation of mitochondrial proteins in soleus type IIa fibers reflects increased numbers of mitochondria. A**) Volcano plot comparing protein fold-changes (log_2_-scale) in soleus type I and type IIa fibers (Table S3). Colored circles reflect mitochondrial proteins (blue, MitoCarta2.0-annotated) and OXPHOS complex members. **B**) The same volcano plot as in **A**) but showing normalized values for mitochondrial annotated proteins. Respiratory chain complex I, III, IV and V members were selected, and mean intensities were calculated for type I and IIa samples separately. A correction factor was calculated by correlating the average value of type I complex members to the average value of type II members. The mean relative intensity in type I fibers was 0.745 (compared to type IIa).

**Additional file 4: **: **Suppl. Figure 4: Increased fluorescence of tropomyosin-1 stained EDL type IIb compared to TA type IIb myofibers. A**) Co-immunostaining of tropomyosin-1 (TPM1) and myosin-4 in muscle cryosections showing the TA (left to the dashed line) and EDL (right to the dashed line) muscle. Type IIb positive myofibers are marked with “a” (TA) or “b” (EDL). **B**) Quantitative analysis of the TPM1 fluorescence signal in type IIb positive fibers of the TA or the EDL muscle. Significance was tested by two-sided t-testing (*n* = 40). **C**) Co-immunostaining of titin (TTN) and myosin-4 in muscle cryosections showing the TA (left to the dashed line) and EDL (right to the dashed line) muscle. Type IIb positive myofibers are marked with “a” (TA) or “b” (EDL). **B**) Quantitative analysis of the TTN fluorescence signal in type IIb positive fibers of the TA or the EDL muscle. Significance was tested by two-sided t-testing (*n* = 30)

**Additional file 5 **: **Suppl. Figure 5: In-vivo SILAC reveals similar Lys-6 labeling after 14 days of MYH isoforms and muscle groups. A**) Lys-6 labeling of type I muscle fibers from the soleus ranged from 10% to 80%, with the majority of proteins exhibiting 30–-40% Lys-6 labeling. Notably, albumin (ALB) and transferrin (TF) exhibited ~70% Lys-6 labeling; representative SILAC pairs for the albumin peptide TPVSEHVTK substantiated this high Lys-6 labeling. Grey circles indicate the Lys-0 peptide; red circles the Lys-6-labeled peptide. **B**) On average, liver proteins exhibited higher Lys-6 labeling rates ranging from ~60–85%. Albumin and transferrin had the same Lys-6 labeling rates in liver tissues as in muscle fibers, suggesting that albumin and transferrin are taken up by muscle fibers via the blood system. The same albumin peptide (TPVSEHVTK) showed similar Lys-6 labeling in muscle fibers and liver tissues. **C**) Selected SILAC pairs of muscle fiber proteins. The grey circle marks a non-labeled “light” Lys-0 peptide and the red circle illustrates the newly synthesized “heavy” Lys-6 peptide. Titin exhibited very similar Lys-6 incorporation rates in type I and IIa fibers (~31%), whereas Myomesin 2 had a lower incorporation rate in type I fibers (~38%) than type IIa fibers ( ~46%). Conversely, isocitrate-dehydrogenase 3b showed a higher Lys-6 labeling rate in type I ( ~54%) than type IIa fibers ( ~44%).

**Additional file 6 **: **Table S1:** Data for comparison of whole muscle and single fiber proteomes related to Fig.ure 1. **S1_proteingroups.txt:** Protein groups output table of whole muscle and single muscle fiber (*n* = 48) lysates for Venn diagram representation. **S1_ident_protgroups:** Number of identified proteingroups in 1x, 5x, 10x, 20x, 48x and 96x fibers as well as whole muscle lysate, related to Fig.ure 1d. **S1_Proteinranking:** Ranking of protein intensities, identified in muscle fibers (*n* = 48) and whole muscle lysates, related to Figure 1c. **S1_GO-enrichment:** GO-annotation enrichment of protein groups only identified in whole muscle lysates or single muscle fibers (48x), related to Figure 1a

**Additional file 7 **: **Table S2:** Data for short LC-MS gradients related to Suppl. Figure 1. **S2_AbsProtCopyNr:** Absolute protein copy numbers per muscle fiber nucleus either calculated using the Proteomics Dynamic Range Standard (UPS2) kit or the proteomic ruler approach, related to Suppl. Figure 1A-B. **S2_Identifications-1-7:** Peptide and protein groups output table of different LC-MS gradients measured with the Easy nLC II-LTQ-Orbitrap Discovery system, related to Suppl. Figure 1C **–** E. **S2_proteingroups_CAPvsnLC:** Protein groups output table of single fiber ProFiT runs using the Easy nLC II - LTQ Orbitrap and capillary LC/quadrupole Orbitrap system, related to Suppl. Figure 1F. **S2_peptides_CAPvsnLC:** Peptide output table of single fiber ProFiT runs using the Easy nLC II - LTQ Orbitrap and capillary LC/quadrupole Orbitrap system, related to Suppl. Figure 1G. **S2_CarryOver:** List of log_2_ LFQ intensities of short gradients with six muscle fibers and blank runs to test for potential carryover, related to Suppl. Figure 1H. **S2_Top5MyHCPeptides:** List of MyHC peptides analyzed with ProFit with its mass, retention time and peptide scores measured on an Easy nLC II - LTQ Orbitrap or a capillary LC/quadrupole Orbitrap system, related to Suppl. Figure 1I-J

**Additional file 8 **: **Table S3:** Fiber profiling data and protein groups output table of type I and type IIa fibers. **S3_ProFiTvsIHC:** Summary of fiber-type profiling of the soleus and EDL using either ProFiT or immunohistochemistry, related to Figure 2a**. S3_protein groups ProFiT:** Protein groups output table of single muscle fiber proteomes analyzed by ProFiT, related to Figure 2a. **S3_Summary:** MaxQuant summary.txt table of the proteomics analysis of pooled soleus type I and type IIa muscle fiber, related to Figure 2b. **S3_protein groups_ident:** Protein groups output table for proteins identified in type I and IIa fibers, related to Figure 2b. **S3_protein groups_quant:** Protein groups output table for type I and IIa quantification, related to Figure 2b. **S3 _1DannotEnrich:** 1D protein annotation of proteins identified in soleus type I and IIa fibers, related to Fig.ure 2c. **S3_TMEM_IHC:** Quantification of TMEM65 signal intensities on soleus and EDL cross-sections, related to Suppl. Figureure 2E-I. **S3_CSP1_IHC:** Quantification of CPS1 signal intensities on soleus cross-sections related to Figure 2d-e. **S3_protein groups_MTNorm:** Protein groups output table of type I and IIa quantification with OXPHOS-normalized mitochondrial protein abundances, related to Suppl. Figureure3 A**-**B.

**Additional file 9 **: **Table S4:** Analysis of type IIb fibers from the EDL, TA, and Gast. **S4_Summary:** MaxQuant summary.txt table of the proteomics analysis in pooled type IIb muscle fibers of the EDL, GAST, and TA, related to Fig.ure 3a. **S4_protein groups_ident:** Protein groups output table of identified proteins in type IIb fibers of the EDL, TA, and GAST, related to Fig.ure 3a. **S4_protein groups_quant:** Protein groups output table of quantified proteins in type IIb fibers of the EDL, TA, and GAST, related to Figure 3a. **S4_Fisher:** Summary of Fisher’s exact tests of type IIb fibers, related to Figure 3b. **S4_IHC:** Quantification of TPM1, DES and TTN signal intensities on soleus cross-sections related to Figure 3d-e and Suppl. Figure 3A-D

**Additional file 10 **: **Table S5:** Analysis of Lys-6 incorporation rates into muscle fiber types. **S5_ProFiT:** Protein groups output table of muscle fibers analyzed with ProFiT, related to Figure 4b-c. **S5_ProtIncorp_Ttest:***T*-test of Lys-6 incorporation rates for proteins in different muscle fiber types analyzed with ProFiT, related to Figure 4b. **S5_MyHCIncorp_Ttest***T*-test of Lys-6 incorporation rates into MyHC isoforms in different muscle fiber types analyzed with ProFiT, related to Figure 4c. **S5_protein groups_HL-ratios:** Protein group output table of H/L ratios (Lys6/Lys0) in pooled muscle fiber types, related to Figure 4d-l. **S5_protein groups_IncorpRates:** Protein group output table of Lys-6 incorporation rates in pooled muscle fiber types, related to Figure 4d-l. **S5_Complex I_Testing:***T*-test of Lys-6 incorporation rates into respiratory chain complex I modules, related to Figure 4l. **S5_HL ratio Liver:** protein groups output table of H/L ratios of Lys-6 to Lys-0 in liver tissue, related to suppl. Figure 4B. **S5_protein groups_Lys6_Tissue:** Protein groups output table of Lys-6 incorporation rates in different mouse tissues, related to Figure 4m. **S5_T-test_ComplexIV:***T*-test of Lys-6 incorporation rates into respiratory chain complex IV subunits, related to Figure 4m

**Additional file 11 **: **Table S6:** Muscle fiber type-specific analysis of phosphosites. **S6_Phospho(STY)_Ident:** Phospho (STY) sites output table of identified phosphorylation sites in soleus type I and type IIa fibers, related to Figure 5b-f. **S6_Phospho(STY)_Quant:** Phospho (STY) sites output table of quantified phosphorylation sites in soleus type I and type IIa fibers, related to Figure 5b-f. **S6_proteingroups_Ident:** Fiber type-specific phosphorylation patterns on the giant protein titin (TTN), related to Figure 5f. **S6_proteingroups_Quant:** Protein groups output table of protein intensities in soleus type I and IIa fibers, related to Figure 5c-e

## Data Availability

The mass spectrometry proteomics data have been deposited to the ProteomeXchange Consortium via the PRIDE partner repository with the dataset identifier PXD017755.

## Abbreviations

ACN: Acetonitrile
ACTN: Alpha-actinin
AGC: Automatic gain control
COX: Cytochrome c oxidase
DDA: Data-dependent acquisition
DIA: Data-independent acquisition
DMEM: Dulbecco’s modified eagle’s medium
DTT: Dithiothreitol
EDL: Extensor digitorum longus muscle
FA: Formic acid
GAST: Gastrocnemius muscle
GO: Gene ontology
GuHCl: Guanidine hydrochloride
HCD: Higher-energy collisional dissociation
IAA: Iodoacetamide
IDH: Isocitrate dehydrogenase
IT: Injection time
LC-MS: Liquid chromatography–mass spectrometry
Lys-6: 13C6 lysine
MYBPC: Myosin-binding protein C
MyHC/MYH: Myosin heavy chain
MYL: Myosin light chain
NCE: Nominal collisional energy
NDUF: NADH:ubiquinone oxidoreductase
OBSCN: Obscurin
OXPHOS: Oxidative phosphorylation
PBS: Phosphate-buffered saline
ProFiT: Proteomics high-throughput fiber typing
PTM: Posttranslational modification
ROS: Reactive oxygen species
SDS: Sodium dodecyl sulfate
SILAC: Stable isotope labeling of amino acids in cell culture
TA: Tibialis anterior muscle
TBS: Tris-buffered saline
TCA: Tricarboxylic acid cycle
TFA: Trifluoroacetic acid
TiO_2_: Titanium dioxide
TMEM: Transmembrane protein
UPS2: Proteomics dynamic range standard
