# Supplementary material for: High-throughput proteomics fiber typing (ProFiT) for comprehensive characterization of single skeletal muscle fibers
Source: Skelet Muscle. 2020 Mar 23;10:7. doi: 10.1186/s13395-020-00226-5 (PMC7087369; doi:10.1186/s13395-020-00226-5)

Suppl. Figure 1: LC-MS gradient optimization and identification of unique peptides from different MYH isoforms

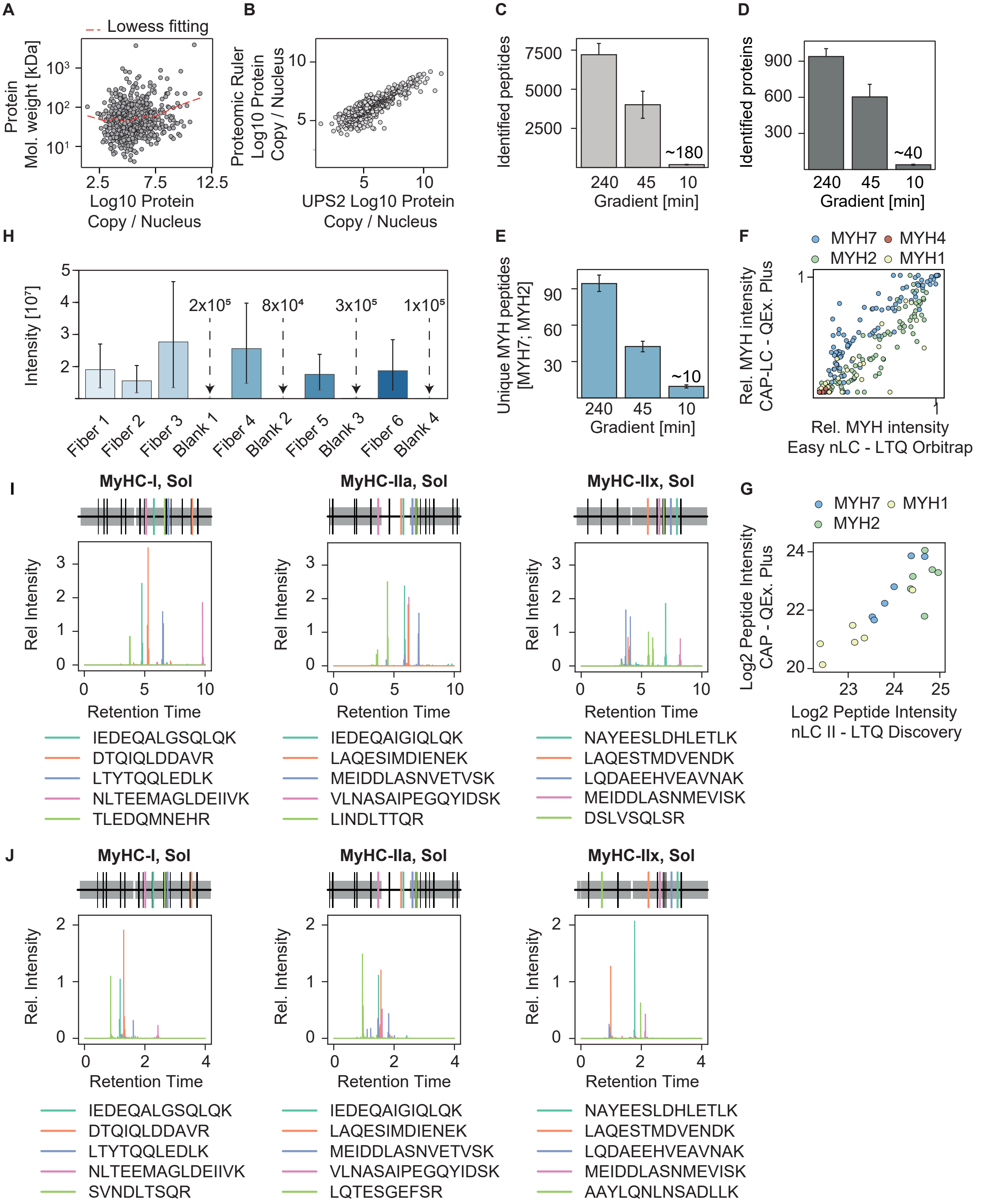

Supplement: Supplementary file 1 — Additional file 1 : Suppl. Figure 1: LC-MS gradient optimization and identification of unique peptides from different MYH isoforms. A) Calculation of absolute copy numbers per nucleus by spike-in of the Proteomics Dynamic Range Standard (UPS2) indicates the high dynamic range of muscle fibers. The detection limit was estimated to be ~200 protein copies per muscle fiber nucleus. Conversely, the most abundant protein MYH1 had a copy number of 3x10^11^ copies per nucleus. The dashed red line indicates a local regression (locally weighted scatterplot smoothing). B) Absolute protein copy numbers calculated with the UPS2 kit or the Proteomic Ruler approach. C-E) Bar diagrams indicating identified C) peptides, D) proteins and E) unique MYH peptides identified from 23 single muscle fibers of the soleus muscle using a linear ion trap instrument. LC-MS gradients ranged from 10 min to 240 min. F) Muscle fiber type distribution between a linear ion trap Orbitrap (LTQ Orbitrap) coupled to an Easy nLC II and a quadrupole Orbitrap (QExactive HF-X) instrument coupled to a Dionex Ultimate 3000 UHPLC. Colored dots indicate the predominant MYH protein in the respective muscle fiber. G) Comparison of the top 6 MYH1, MYH2 and MYH7 peptide intensities identified on an Easy nLC II-LTQ Orbitrap setup and a CAP-LC-QExactive HF-X setting. H) Measurement of peptide carry-over after a ProFiT run using 10 min chromatographic gradients. No detectable carryover of peptides was observed between samples of single muscle fibers and blank runs. The measured intensities of blank runs were 2-3 orders of magnitude lower compared to the single muscle fiber samples and virtually no MYH peptides were detectable in blank runs. I-J) Top five most abundant unique MYH peptides from three different isoforms were identified using either I) the Easy nLC-II-LTQ Orbitrap or J) the CAP-LC-QExactive HF-X setting. The peptide position within each protein is illustrated in the upper panel. The exact position and retentio [file 13395_2020_226_MOESM1_ESM.pdf]
