# Supplementary figures and images for: High-throughput proteomics fiber typing (ProFiT) for comprehensive characterization of single skeletal muscle fibers
Source: Skelet Muscle. 2020 Mar 23;10:7. doi: 10.1186/s13395-020-00226-5 (PMC7087369; doi:10.1186/s13395-020-00226-5)

**Suppl. Figure 2: Systematic view of protein changes between slow and fast muscle fibers**

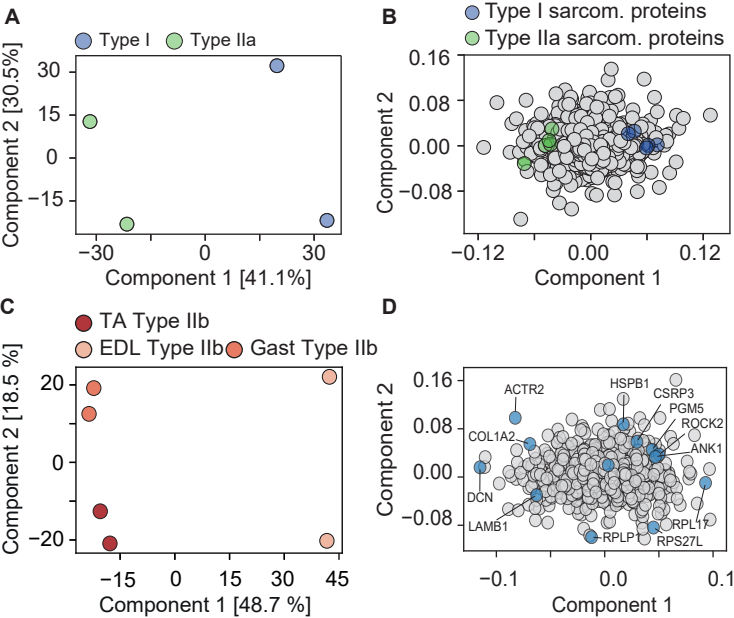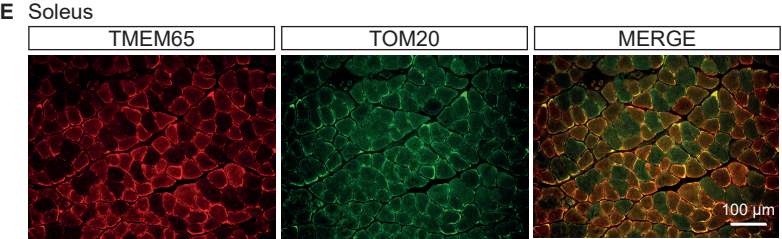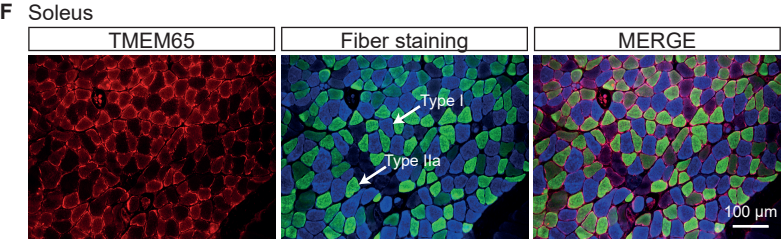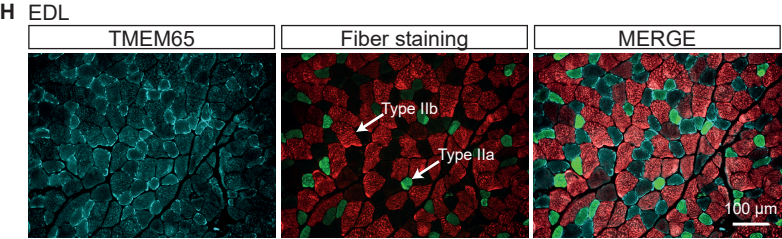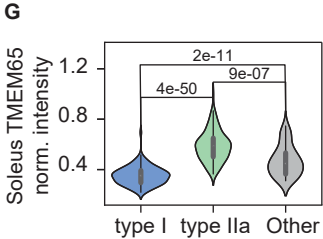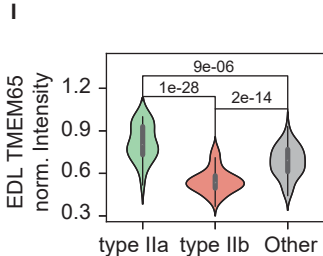

Supplement: Supplementary file 2 — Additional file 2 : Suppl. Figure 2: Systematic view of protein changes between slow and fast muscle fibers. A) Principal component analysis (PCA) revealed a clear separation of type I and type IIa fibers from the soleus muscle and B) indicates the main components responsible for the separation (green for type IIa and blue for type I). C) Similarly, PCA clearly confirmed the separation of protein intensities for type IIb fibers from the EDL, TA, and Gast. D) Blue labeled circles represent the main components responsible for the separation. E) Overview of co-immunostaining of soleus cross-sections with antibodies that recognize TMEM65 and the mitochondrial outer membrane protein TOM20. F) Co-immunostaining of soleus cross-sections with TMEM65 and antibodies that bind specifically to slow MYH7 (type) and fast MYH2 (type IIa) isoforms. G) Quantitative analysis of TMEM65 fluorescence signal intensities in different fiber types in the soleus muscle. Significance was tested by two-sided t-tests (n = 297). H) EDL cross-sections immunostained with TMEM65, MYH2, and MYH4. I) Quantitative analysis of the TMEM65 fluorescence signal in EDL myofibers. Significances were tested by two-sided t-tests (n = 200) [file 13395_2020_226_MOESM2_ESM.pdf]

**Suppl. Figure 5: *in vivo* SILAC Lys-6 labeling rates after 14 days in MYH isoforms and muscle groups**

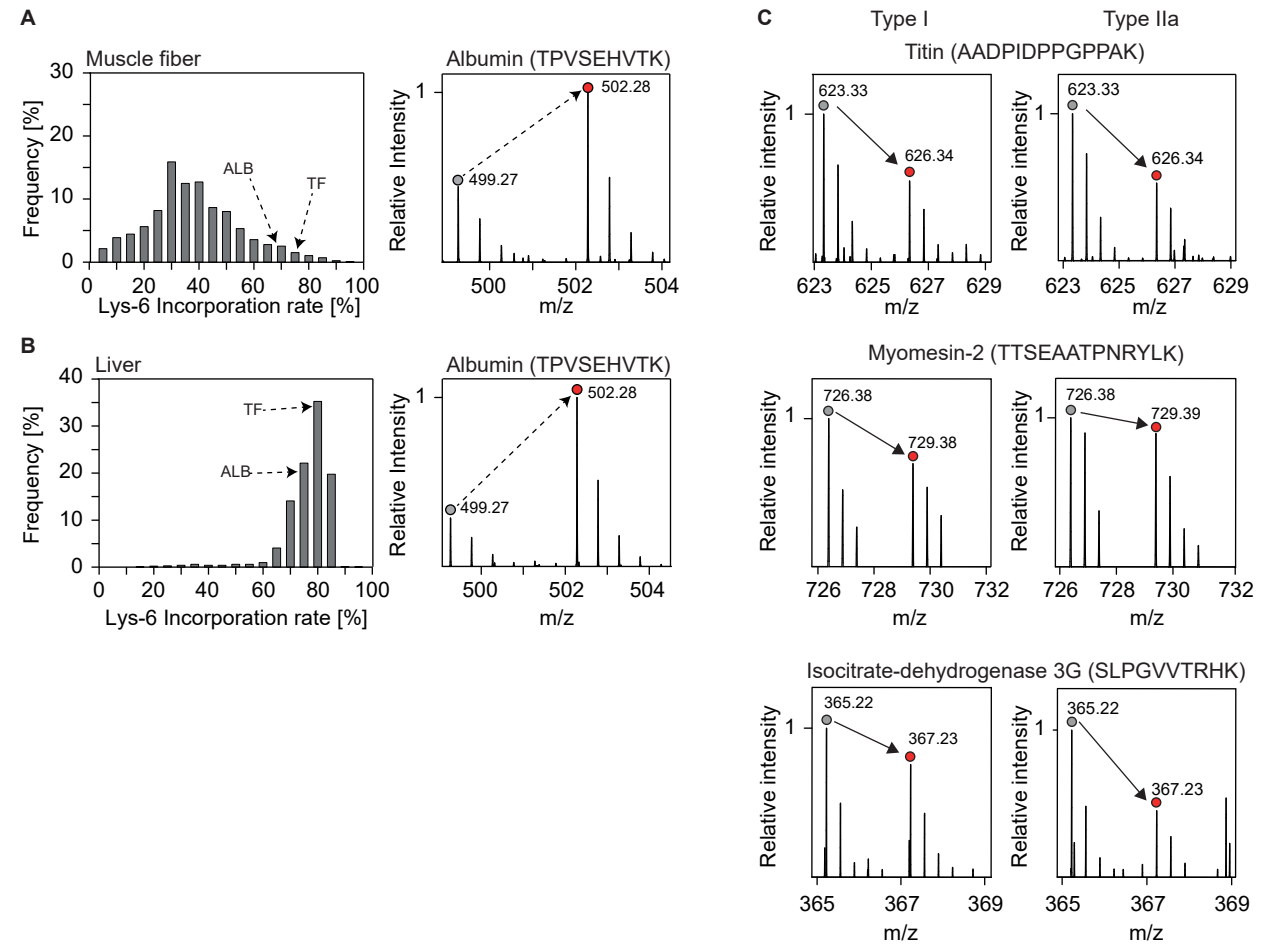

Supplement: Supplementary file 5 — Additional file 5 : Suppl. Figure 5: In-vivo SILAC reveals similar Lys-6 labeling after 14 days of MYH isoforms and muscle groups. A) Lys-6 labeling of type I muscle fibers from the soleus ranged from 10% to 80%, with the majority of proteins exhibiting 30–-40% Lys-6 labeling. Notably, albumin (ALB) and transferrin (TF) exhibited ~70% Lys-6 labeling; representative SILAC pairs for the albumin peptide TPVSEHVTK substantiated this high Lys-6 labeling. Grey circles indicate the Lys-0 peptide; red circles the Lys-6-labeled peptide. B) On average, liver proteins exhibited higher Lys-6 labeling rates ranging from ~60–85%. Albumin and transferrin had the same Lys-6 labeling rates in liver tissues as in muscle fibers, suggesting that albumin and transferrin are taken up by muscle fibers via the blood system. The same albumin peptide (TPVSEHVTK) showed similar Lys-6 labeling in muscle fibers and liver tissues. C) Selected SILAC pairs of muscle fiber proteins. The grey circle marks a non-labeled “light” Lys-0 peptide and the red circle illustrates the newly synthesized “heavy” Lys-6 peptide. Titin exhibited very similar Lys-6 incorporation rates in type I and IIa fibers (~31%), whereas Myomesin 2 had a lower incorporation rate in type I fibers (~38%) than type IIa fibers ( ~46%). Conversely, isocitrate-dehydrogenase 3b showed a higher Lys-6 labeling rate in type I ( ~54%) than type IIa fibers ( ~44%). [file 13395_2020_226_MOESM5_ESM.pdf]
