# Supplementary material for: High-throughput proteomics fiber typing (ProFiT) for comprehensive characterization of single skeletal muscle fibers
Source: Skelet Muscle. 2020 Mar 23;10:7. doi: 10.1186/s13395-020-00226-5 (PMC7087369; doi:10.1186/s13395-020-00226-5)

Suppl. Figure 3: Overrepresentation of mitochondrial proteins in soleus type IIa reflects increased numbers of mitochondria

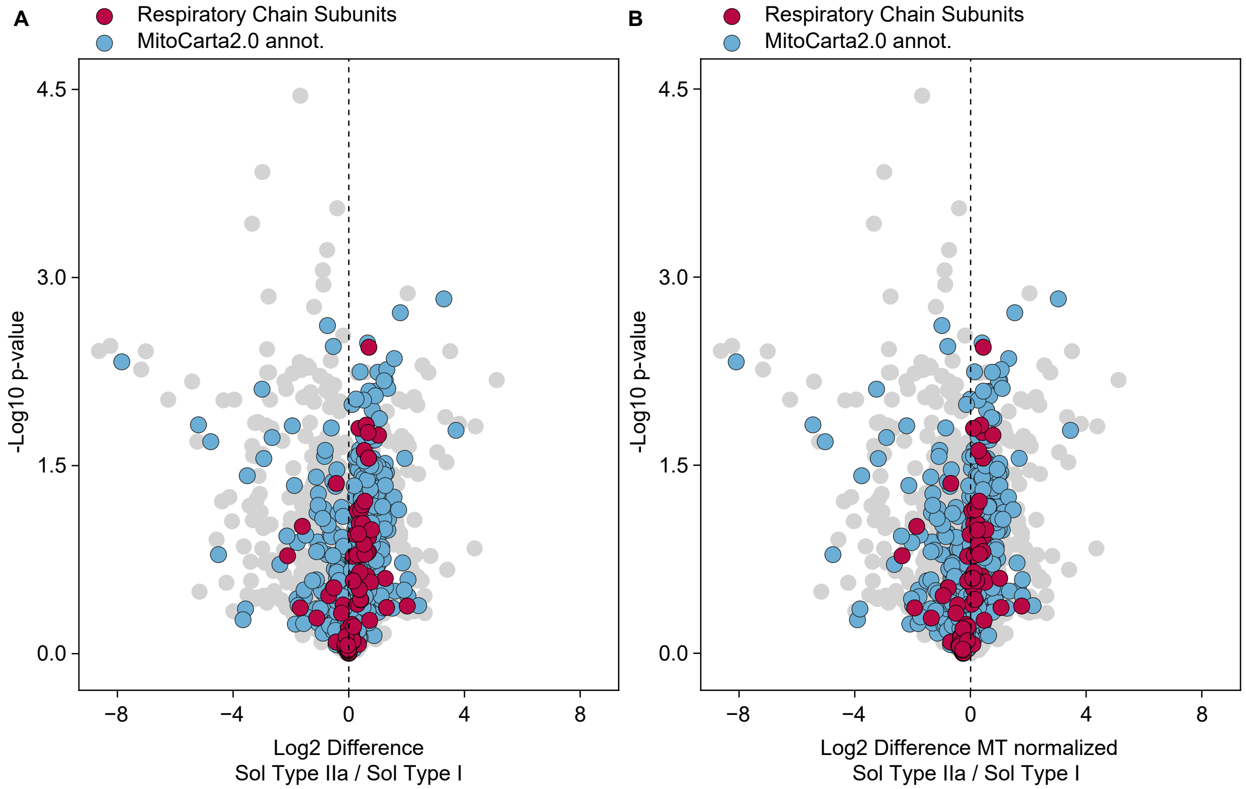

Supplement: Supplementary file 3 — Additional file 3: : Suppl. Figureure 3: Overrepresentation of mitochondrial proteins in soleus type IIa fibers reflects increased numbers of mitochondria. A) Volcano plot comparing protein fold-changes (log2-scale) in soleus type I and type IIa fibers (Table S3). Colored circles reflect mitochondrial proteins (blue, MitoCarta2.0-annotated) and OXPHOS complex members. B) The same volcano plot as in A) but showing normalized values for mitochondrial annotated proteins. Respiratory chain complex I, III, IV and V members were selected, and mean intensities were calculated for type I and IIa samples separately. A correction factor was calculated by correlating the average value of type I complex members to the average value of type II members. The mean relative intensity in type I fibers was 0.745 (compared to type IIa). [file 13395_2020_226_MOESM3_ESM.pdf]
