# Supplementary material for: High-throughput proteomics fiber typing (ProFiT) for comprehensive characterization of single skeletal muscle fibers
Source: Skelet Muscle. 2020 Mar 23;10:7. doi: 10.1186/s13395-020-00226-5 (PMC7087369; doi:10.1186/s13395-020-00226-5)

Suppl. Figure 4: Increased fluorescence of tropomyosin-1 stained EDL type IIb compared to TA type IIb myofibers

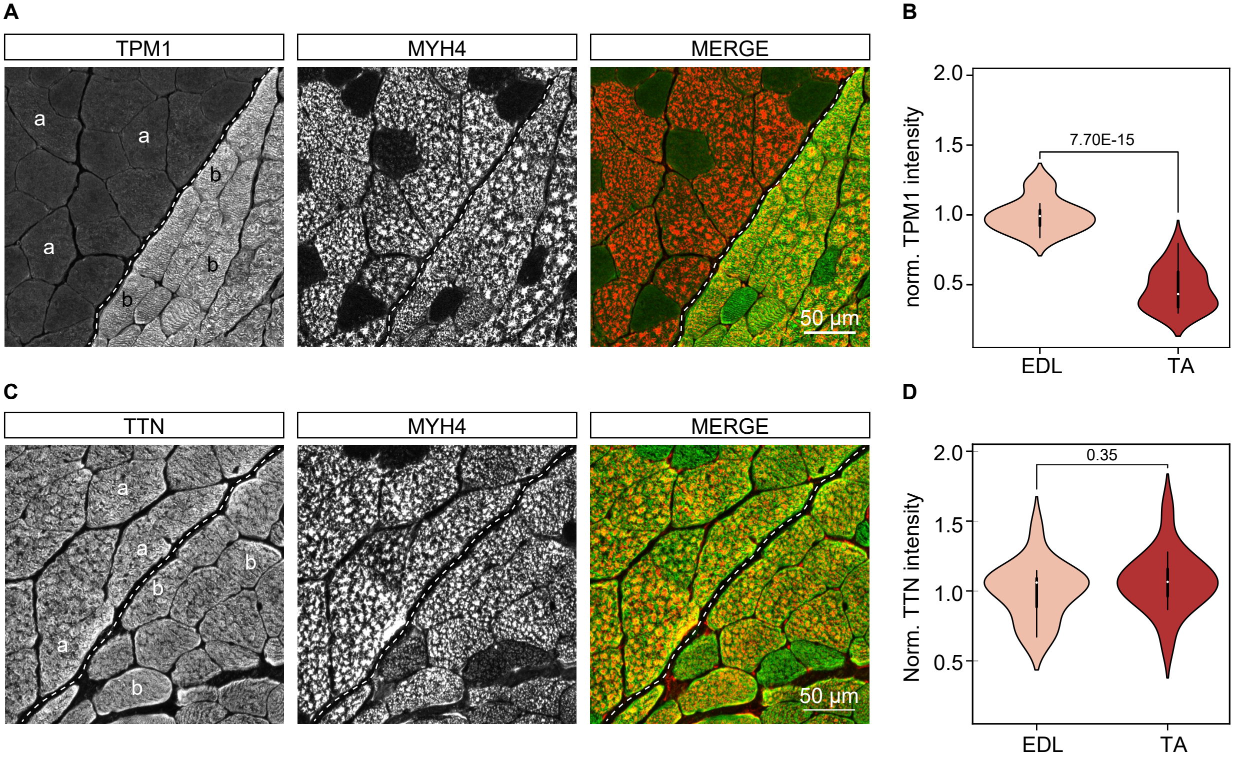

Supplement: Supplementary file 4 — Additional file 4: : Suppl. Figure 4: Increased fluorescence of tropomyosin-1 stained EDL type IIb compared to TA type IIb myofibers. A) Co-immunostaining of tropomyosin-1 (TPM1) and myosin-4 in muscle cryosections showing the TA (left to the dashed line) and EDL (right to the dashed line) muscle. Type IIb positive myofibers are marked with “a” (TA) or “b” (EDL). B) Quantitative analysis of the TPM1 fluorescence signal in type IIb positive fibers of the TA or the EDL muscle. Significance was tested by two-sided t-testing (n = 40). C) Co-immunostaining of titin (TTN) and myosin-4 in muscle cryosections showing the TA (left to the dashed line) and EDL (right to the dashed line) muscle. Type IIb positive myofibers are marked with “a” (TA) or “b” (EDL). B) Quantitative analysis of the TTN fluorescence signal in type IIb positive fibers of the TA or the EDL muscle. Significance was tested by two-sided t-testing (n = 30) [file 13395_2020_226_MOESM4_ESM.pdf]
